# CD146 regulates the stemness and chemoresistance of hepatocellular carcinoma via JAG2-NOTCH signaling

**DOI:** 10.1038/s41419-025-07470-x

**Published:** 2025-03-03

**Authors:** Bing Yan, QiuYu Lu, TianMing Gao, KunQing Xiao, QianNi Zong, HongWei Lv, GuiShuai Lv, Liang Wang, ChunYing Liu, Wen Yang, GuoQing Jiang

**Affiliations:** 1 Department of Hepatobiliary Surgery, Northern Jiangsu People’s Hospital, Yangzhou, 225000 China; 2 Department of Hepatobiliary Surgery, Northern Jiangsu People’s Hospital Affiliated to Yangzhou University, Yangzhou, 225000 China; 3 Department of General Surgery, Pingxiang People’s Hospital, Pingxiang, 337000 China; 4 International Cooperation Laboratory on Signal Transduction, Eastern Hepatobiliary Surgery Institute, Naval Medical University (Second Military Medical University), Shanghai, 200438 China; 5 National Center for Liver Cancer, Naval Medical University (Second Military Medical University), Shanghai, 201805 China; 6 Shanghai Key Laboratory of Hepatobiliary Tumor Biology, Shanghai, 200438 China; 7 Key Laboratory of Signaling Regulation and Targeting Therapy of Liver Cancer, Ministry of Education, Shanghai, 200438 China

**Keywords:** Cancer stem cells, Cancer stem cells

## Abstract

CD146 plays a key role in cancer progression and metastasis. Cancer stem cells (CSCs) are responsible for tumor initiation, drug resistance, metastasis, and recurrence. In this study, we explored the role of CD146 in the regulation of liver CSCs. Here, we demonstrated that CD146 was highly expressed in liver CSCs. CD146 overexpression promoted the self-renewal ability and chemoresistance of Hepatocellular Carcinoma (HCC) cells in vitro and tumorigenicity in vivo. Inversely, knockdown of CD146 restrained these abilities. Mechanistically, CD146 activated the NF-κB signaling to up-regulate JAG2 expression and activated the Notch signaling, which resulted in increased stemness of HCC. Furthermore, JAG2 overexpression restored the Notch signaling activity, the stemness, and chemotherapeutic resistance caused by CD146 knockdown. These results demonstrated that CD146 positively regulates HCC stemness by activating the JAG2-NOTCH signaling. Combined targeting of CD146 and JAG2 may represent a novel therapeutic strategy for HCC treatment.

## Introduction

Hepatocellular carcinoma (HCC) is the main pathological type of primary liver cancer, the sixth most common cancer, and the third leading cause of cancer-related mortality worldwide [1]. Recent data showed that HCC is the second most prevalent malignancy in China, with more than 300,000 deaths from liver cancer were reported in 2020 alone [2].

While surgical resection is a treatment option for HCC, nevertheless, the rate of recurrence after liver resection is up to 70% at 5 years, and recurrence is associated with poor outcomes [3]. Like most solid tumors, HCC tumors are associated with the presence of cancer stem cells (CSCs), which exhibit self-renewal, infinite proliferation, and tumorigenic characteristics [4]. An increasing body of evidence has suggested that cancer stemness contributes to carcinogenesis, tumor relapse, and chemoresistance, suggesting that targeting CSCs may be a breakthrough in the search for treatments for HCC [5–7]. Therefore, the identification of molecules and regulatory mechanisms involved in HCC stemness is critical.

Some studies have demonstrated that CSCs share the same regulatory genes and signaling pathways with embryonic and tissue stem cells [8]. For example, the Notch, Hedgehog and Wnt signaling pathways, which play key roles in embryonic development, are involved in the maintenance of CSC phenotypes [4, 9]. Notch signaling plays a significant role in cancer progression [10]. SMAD7 overexpression promotes HCC in mice and humans by activating the YAP/Notch cascade [11]. Overexpression of ABL1 regulates tumor development by regulating the Notch1 pathway [12]. Additionally, one study showed that persistent Notch signaling drives the development of tumors in chronic liver diseases [13]. Furthermore, numerous studies have indicated that the activation of the Notch signaling pathway promotes stemness [14–16]. These findings demonstrate that the Notch signaling pathway contributes to maintaining cancer stemness and promoting tumorigenesis.

CD146, also known as MUC18 or MCAM, was first identified in malignant melanoma and plays an important role in driving melanoma progression and metastasis [17]. Previous studies have shown that CD146 participates in cell-cell adhesion, inflammation, and angiogenesis [18–20], and it accelerates tumor progression by promoting angiogenesis and metastasis [21, 22]. In our previous study, we demonstrated that CD146 promotes metastasis and predicts the poor prognosis of HCC [23]. Accumulating evidence has shown that CD146 is a mesenchymal stem cell marker [24, 25]. Ghanekar et al. confirmed that CD146 plays an important role in liver cancer as a marker of tumor stem cells or progenitor cells [26]. Moreover, CD146 was shown to contribute to the cancer stemness phenotype in Epidermal Growth Factor Receptor-Tyrosine Kinase Inhibitor (EGFR-TKI) - resistant lung cancer cells [27]. Yawata et al. reported that overexpression of CD146 accelerates the growth of glioma stem cells and contributes to the self-renewal ability of glioma stem cells [28]. CD146 induces epithelial-mesenchymal transformation, which promotes cancer stem cell–like transition and enhances cell migration in breast cancer [29]. Taken together, these studies demonstrate that CD146 promotes the stemness of cancer cells. However, no studies have explored how CD146 regulates the stemness of HCC cells through the Notch signaling pathway.

In order to explore the role of CD146 in regulating the stemness of HCC cells, we conducted related experiments. Exhilaratingly, we found that CD146 is highly expressed in liver CSCs and the mechanisms by which CD146 upregulates stemness in HCC cells.

## Materials and methods

### Human specimens

HCC samples were obtained from 101 patients with HCC at the Eastern Hepatobiliary Surgery Hospital, Shanghai, China. Informed consent was obtained from all patients. Ethical approval for the study was obtained from the Ethical Committee of the Second Military Medical University (SMMU).

### Cell lines and cell culture

Human liver cancer cell lines, including CSQT-2, PLC/PRF/5, Huh7, Hep3B, MHCC97H and LM3 were provided by Chinese Academy of Sciences Stem Cell Bank, Shanghai, China. The cell lines were cultured in DMEM (Gibco, USA) supplemented with 10% fetal bovine serum (FBS), 100 units/mL penicillin, 100 mg/mL streptomycin and 25 µg/mL amphotericin B for approximately one week in a humidified incubator containing 5% CO_2_ at 37 °C. Stable liver cancer cell lines were obtained for subsequent study.

### Antibodies and reagents

Antibodies for western blotting included mouse anti-β-Actin (1:1000), mouse anti-GADPH (Proteintech, Cat#60004-1-IG, 1:1000), rabbit anti-CD146 antibody (Abcam, Cat#ab75769, 1:1,000), rabbit anti-Jagged2 (JAG2) (Cell Signaling Technology, Cat#2210, 1:1,000), rabbit anti-Notch1 (NOTCH1) (Proteintech, Cat#20687-1-AP, 1:1,000), and rabbit anti-HES1 (ABclonal, Cat#A0925, 1:1,000). Antibodies for IHC included rabbit anti-CD133 (Abcam, Cat#A0219, 1:100), rabbit anti-Oct-4 (Abcam, Cat#A7920, 1:100), rabbit anti-JAG2 (Origene, Cat#TA351308,1:200) and rabbit anti-Ki67 (Proteintech, Cat#27309-1-AP,1:100), and rabbit anti-CD146 (Abcam, Cat#ab75769, 1:500). The Notch inhibitor RO4929097 (S1575) and NF-kB inhibitor QNZ (EVP4593) (S4902) were obtained from Selleck.

### Quantitative real-time PCR

TRIzol reagent (Invitrogen) was used to extract total RNA from cells and tissue. Reverse transcription was performed using Superscript III RT (Invitrogen) and relevant random primers following the manufacturer’s instructions. Quantitative real-time PCR was performed using the SYBR Green PCR Master Mix (Applied Biosystems) on the ABI PRISM 7300HT Sequence Detection System (Applied Biosystems). β-Actin was used as a control for normalization. Primer sequences are listed in Table 1.

**Table 1:** Primer sequences information in the study.

Primer name	Primer sequence (Human)
CD146	F: AGCTCCGCGTCTACAAAGC R: CTACACAGGTAGCGACCTCC
Oct-4	F: CTGGGTTGATCCTCGGACCT R: CCATCGGAGTTGCTCTCCA
EpCAM	F: AATCGTCAATGCCAGTGTACTT R: TCTCATCGCAGTCAGGATCATAA
Nanog	F: TTTGTGGGCCTGAAGAAAACT R: AGGGCTGTCCTGAATAAGCAG
Notch1	F: GAGGCGTGGCAGACTATGC R: CTTGTACTCCGTCAGCGTGA
JAG1	F: GTCCATGCAGAACGTGAACG R: GCGGGACTGATACTCCTTGA
JAG2	F: TGGGCGGCAACTCCTTCTA R: GCCTCCACGATGAGGGTAAA
DLL1	F: GATTCTCCTGATGACCTCGCA R: TCCGTAGTAGTGTTCGTCACA
HES1	F: TCAACACGACACCGGATAAAC R: GCCGCGAGCTATCTTTCTTCA
HEY1	F: GTTCGGCTCTAGGTTCCATGT R: CGTCGGCGCTTCTCAATTATTC
β-Actin	F: CATGTACGTTGCTATCCAGGC R: CTCCTTAATGTCACGCACGAT

### Western blotting

Cells and tissues were lysed with cell lysis buffer (Beyotime Biotechnology) at 4°C for 15 min. Protein concentrations were determined using the bicinchoninic acid (BCA) assay (Thermo Scientific). Proteins samples were separated by SDS-polyacrylamide gel electrophoresis and transferred to NC membranes. The membranes were blocked in TBST with 5% skim milk for 1 h at room temperature, washed in TBST three times, and incubated with specific primary antibodies overnight at 4°C. After washing with TBST three times, the membranes were incubated with fluorescein-conjugated secondary antibodies for 1 h at room temperature.

### Lentivirus production and infection

Lentivirus for overexpression of CD146 and shRNA targeting CD146 were designed and packaged by Genechem Technology (Shanghai, China). The lentivirus system for JAG2 overexpression was designed and packaged by Genomeditech (Shanghai, China). To generate stable CD146 and JAG2 overexpression cells and shCD146 cells, HCC cells were infected with virus; after 48 h, cells were subjected to puromycin selection. Efficient PLC/PRF/5 shCD146#1 and shCD146#2 cell lines were generated via lentiviral infection.

### IHC staining

Human HCC tumor tissues and mouse tumor xenografts were fixed in formalin and embedded in paraffin. IHC staining was performed using the following antibodies: anti-CD133, anti-Oct-4, anti-JAG2, and anti-Ki67 (all at 1:100), and anti-CD146 (1:500). The intensity of staining was scored using Image Scope software (Media Cybernetics, Inc.) as follows: negative staining (score=0), weak staining (score=1), moderate staining (score=2), and strong staining (score=3). Patients were subdivided into two groups by staining: the low expression group (negative or weak staining) and high expression group (moderate or strong staining).

### Sphere formation assays

For sphere formation assays, 3000 HCC-LM3 cells, 5000 Huh7 cells, 5000 PLC/PRF/5 cells, and 10,000 CSQT-2 cells were plated per well in Low Attachment 6-well plates (Corning Incorporated Life Sciences, Acton, MA, USA) and cultured in DMEM supplemented with 10% FBS for 7 days. The number of spheroids was counted using a microscope and representative images were captured. Spheres were collected for qRT-PCR and western blot assays, and cell viability was assessed by Cell Counting Kit 8 (CCK-8) assay.

### Cytoplasmic and nuclear fractions

Cytoplasmic and nuclear proteins were extracted using Nuclear and Cytoplasmic Protein Extraction Kit according to the manufacturer’s instructions (Beyotime, China, Cat#P0028). GAPDH served as the cytoplasmic marker, and Histone H3 served as the nuclear marker.

### Extra limiting dilution assay

Cells were resuspended and seeded in low-attachment plates (5, 10, 50, 100/well for 96 wells, 500/well for 64 wells respectively) with FBS medium for 1 weeks. The number of wells containing spheroids were counted and recorded under a microscope. The website ELDA (http://bioinf.wehi.edu.au/software/elda/) was used to analyze the proportion of cancer stem cells.

### Limiting dilution assay in vivo

For CD146 overexpression cells, 5 × 10^6^ CSQT-2-Ctrl cells, CSQT-2-CD146 cells were subcutaneously injected under the axilla of the right forelimb of male NOD-SCID mice (5 weeks old) (Gempharmatech Co., Ltd, Jiangsu, China), and each group was randomly tested with five mice (n = 5). For CD146 knockdown cells, the limiting dilution assay in vivo was performed, and the 64 NOD-SCID mice were randomly divided into 8 groups, 8 in each group. NOD-SCID mice in each group were injected under the axilla of the right forelimb with the shCD146#2 cells or its control cells (shCtrl) at 1×10^6^, 2×10^5^, 1×10^5^, 2× 10^4^ cells/group respectively. Subcutaneous tumors of the mice were collected after one months. Tumor growth was monitored, and tumor volume was calculated as length×width^2^×0.5. After approximately one month, the tumors were harvested. The website ELDA (http://bioinf.wehi.edu.au/software/elda/) was used to analyze the proportion of cancer stem cells in the mouse subcutaneous tumors and evaluate its sphere formation ability. Animal experiments were approved by the Ethical Committee of the Second Military Medical University and performed following relevant regulations and guidelines.

### Statistical analysis

Data are presented as the mean ± s.d. Two-tailed and Student’s t-test was used to analyze the statistical significance between the two groups. Pearson’s correlation analysis was applied to determine the correlation between two variables. Survival analysis was performed by Kaplan–Meier analysis and log-rank test. p < 0.05 was considered statistically significant.

## Results

### CD146 is highly expressed in liver CSCs

**Fig. 1:**
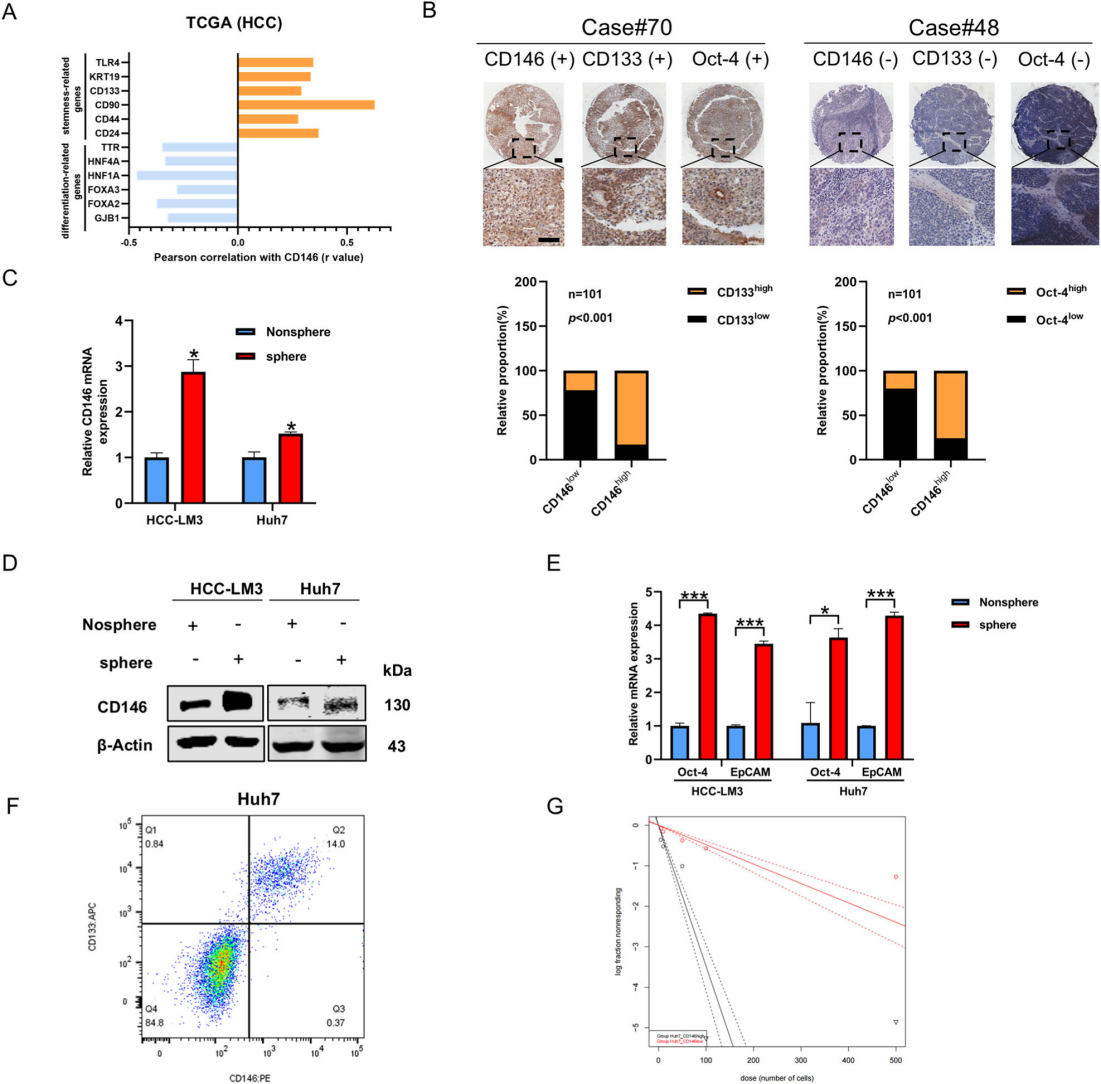
CD146 is highly expressed in liver CSCs. **A** Correlation between stemness-related genes and differentiation-related genes with CD146 in TCGA database (HCC). **B** Up: The correlation analysis of CD146 and CD133 or Oct-4 expressions in human HCC samples. Down: immunohistochemistry analysis of CD146, CD133 and Oct-4 expressions in HCC tissues. Scale bars=100 μm. **C**, **D** The expression of CD146 in tumor spheres generated from HCC cells lines and non-spheres by RT-qPCR and western blotting. **E** The expression of Oct-4 and EpCAM in tumor spheres generated from HCC cells lines and non-spheres by RT-qPCR. **F** Flow cytometry to detect the enrichment of CD133 in Huh7 cells. **G** The Extreme limiting dilution analysis (ELDA) was performed that the sphere formation efficiency of CD146+cells and CD146-cells in Huh7 cells. Data are representative of at least three independent experiments and shown as mean ± s.d (*p < 0.05; ***p < 0.001).

Stemness-related genes are highly expressed in stem cells; the encoded proteins inhibit differentiation-related genes and maintain stemness. By analyzing TCGA database, the results showed that stemness-related genes TLR4 [30] and KRT19 [31] and the CSC markers CD133, CD90, CD44, and CD24 [32] positively correlated with CD146 and the differentiation-related markers including GJB1, FOXA2, FOXA3, HNF1A, HNF4A, and TTR [33] negatively correlated with CD146 in HCC (Fig. 1A). Moreover, CD146 expression was positively correlated with stemness-related genes (such as CD133 and Oct-4) in HCC tissue samples (Fig. 1B). Additionally, Reverse transcription-quantitative PCR (RT-qPCR) showed that CD146 expression was increased in sphere cells (from HCC-LM3 and Huh7 lines) compared with the corresponding adherent cells (Fig. 1C), which was confirmed by western blotting (Fig. 1D). Furthermore, the mRNA expression of stemness-related genes Oct-4 and EpCAM was dramatically elevated in sphere cells compared with the corresponding adherent cells (Fig. 1E). Meanwhile, to demonstrate whether there are separate cell populations based on the expression levels of CD146 in the cell lines used, First, we determined the expression of CD146 in HCC cell lines by Western blotting (Fig. S1). We found that CD146 was highly expressed in Huh7, Hep3B and PLC/PRF/5 cells. Following, the Fluorescence-Activated Cell Sorting (FACS) analyses was performed in Huh7 cells. As shown in (Fig. 1F) that the CSC marker CD133 were enriched in CD146 + /high population, compared to CD146-/low/dim population. The Extreme limiting dilution analysis (ELDA) showed that the sphere formation efficiency of CD146+ cells was better than that of CD146-cells (Fig. 1G). There results suggest that CD146 is highly expressed in liver CSCs.

### CD146 overexpression promotes the stemness of HCC cells

**Fig. 2:**
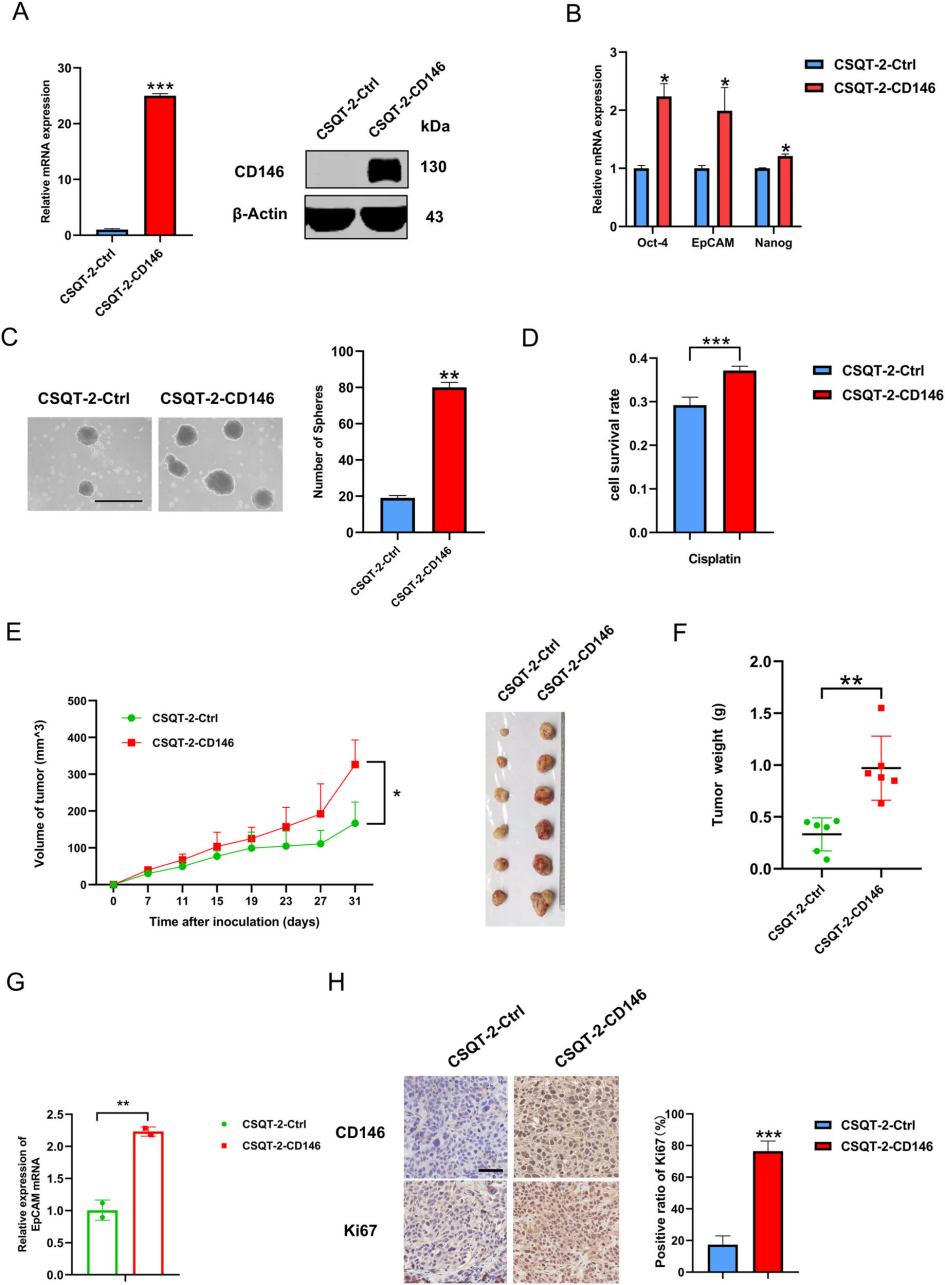
CD146 overexpression promotes the stemness of HCC cells. **A** CD146 were stably overexpressed in CSQT-2 cells by lentiviral transfection. Its expression was assessed at both mRNA and protein levels. **B** RT-qPCR analysis of Oct-4, EpCAM and Nanog in CSQT-2-Ctrl or CSQT-2-CD146 cells. **C** Comparison of sphere formation ability between CSQT-2-Ctrl and CSQT-2-CD146 cells by accessing number of tumor spheres (7 days), Scale bars=120 μm. **D** CSQT-2-Ctrl and CSQT-2-CD146 cells were exposed to cisplatin at the concentration corresponding to its IC_50_ value for 48 h. CCK-8 assay elucidated the cell viability. **E** CSQT-2-Ctrl and CSQT-2-CD146 cells were subcutaneously injected into NOD-SCID mices (CSQT-2-Ctrl n = 5, CSQT-2-CD146 n = 5). Tumors growth curves (Left) and representative tumors (Right) were shown. **F** Tumor weights were measured when mice were sacrificed. **G** RT-qPCR analysis of EpCAM in CSQT-2-CD146 cells-derived tumor tissues compared to CSQT-2-Ctrl cells-derived tumor tissues from mice. **H** Immunohistochemical (IHC) analysis of proliferation marker Ki-67 in CSQT-2-CD146 cells-derived tumor tissues and CSQT-2-Ctrl cells-derived tumor tissues from mice. Representative images (Left), Scale bars = 100 μm and quantification of expression of Ki-67 in three regions of an image (Right) were shown. Data are representative of at least three independent experiments and shown as mean ± s.d. (*p < 0.05; **p < 0.01; ***p < 0.001).

We next examined whether CD146 regulates HCC stemness. First, we stably overexpressed CD146 in CSQT-2 cells with lentiviral vectors, and the induction of CD146 mRNA and protein expressions was verified (Fig. 2A). We next examined the mRNA expression of stemness-related genes such as Oct-4, EpCAM, and Nanog in stable CD146 overexpression cells and control cells. Overexpression of CD146 dramatically elevated the transcription of Oct-4, EpCAM, and Nanog (Fig. 2B). Sphere formation is the basis of the self-renewal ability of CSCs [34]. Sphere formation assays showed that CD146 overexpression cells formed more and larger tumor spheres compared with control cells (Fig. 2C), indicating that CD146 overexpression enhanced the self-renewal capacity of liver CSCs. Meanwhile, the ELDA showed that the sphere formation efficiency of CD146 overexpression cells was better than that of control cells (Fig. S2A). Further, half maximal inhibitory concentration (IC_50_) of cisplatin in CSQT-2 cells was determined, which the measured value was 9.8 μg/ml (Fig. S3). And resistance to chemotherapy drugs experiments were performed with reference to the working concentration determined by the IC_50_. The results showed that CD146 overexpression cells showed greater resistance to cisplatin (Fig. 2D).

In vivo, the overexpression of CD146 remarkably enhanced xenograft tumor growth and weight (Fig. 2E–F). Consistent with the in vitro results, the mRNA expression level of the stemness gene EpCAM was increased in tumors derived from CD146-overexpressing cells compared with the controls (Fig. 2G), and immunohistochemistry (IHC) showed increased expression of the proliferation marker Ki-67 (Fig. 2H). Collectively, these results demonstrated that overexpression of CD146 promotes cancer stemness and chemoresistance in HCC cells.

### CD146 knockdown suppresses the stemness of HCC cells

**Fig. 3:**
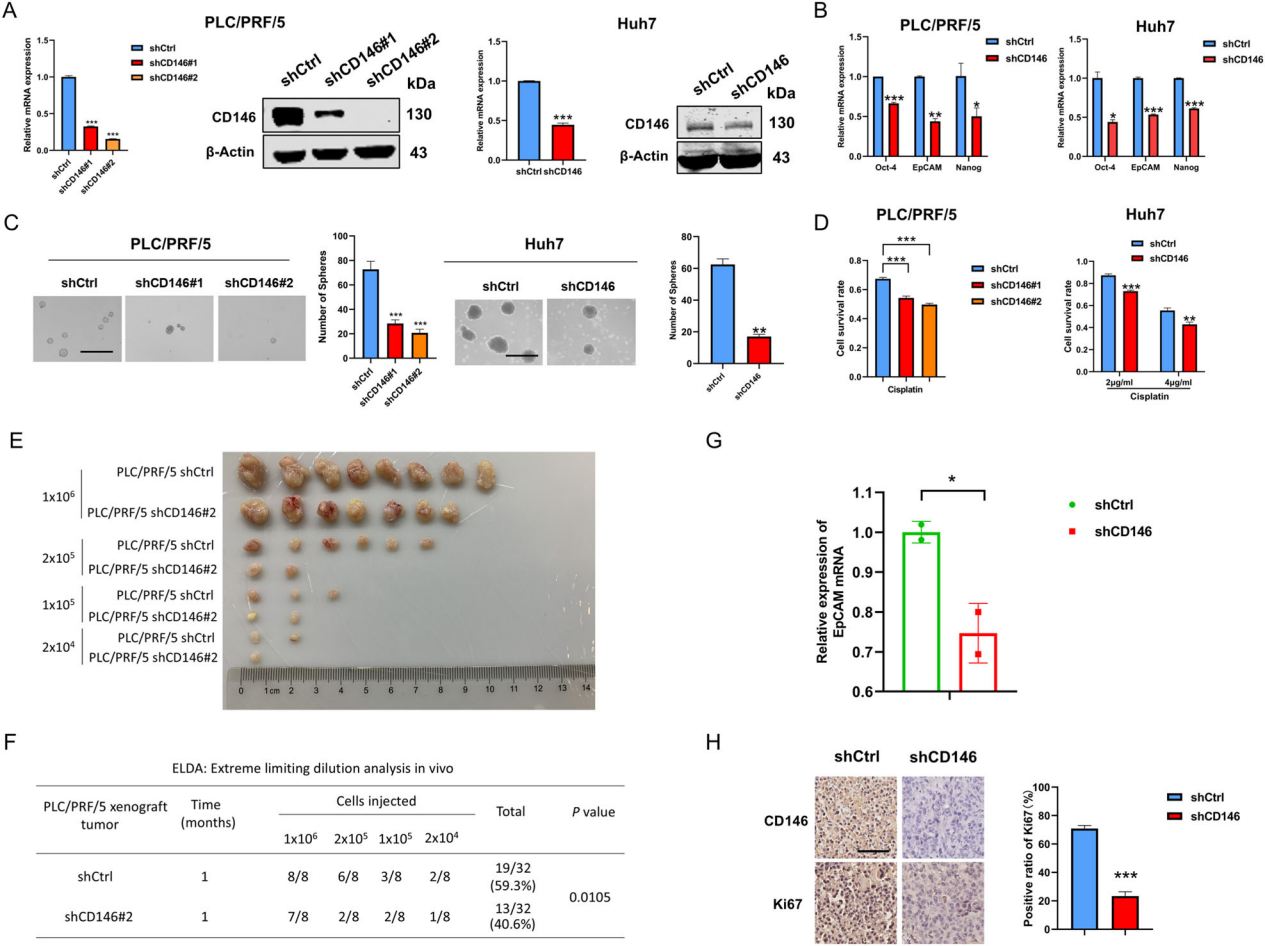
CD146 knockdown suppresses the stemness of HCC cells. **A** CD146 was knocked down in PLC/PRF/5 (Left) and Huh7 (Right) cells with lentivirus shRNA plasmids, CD146 expression was assessed in both mRNA and protein levels. **B** RT-qPCR analysis for expression of Oct-4, EpCAM, Nanog were detected in shCtrl or shCD146 cells. **C** Comparison of sphere formation ability between shCtrl or shCD146 cells by accessing number of tumor spheres (7 days). Scale bars=120 μm. **D** shCtrl and shCD146 cells were exposed to cisplatin at the concentration corresponding to its IC_50_ value for 48 h. CCK-8 assay was carried out to detect cell viability. **E** The number and size of subcutaneous tumors in NOD-SCID mice with varying number of shCtrl and shCD146#2 cells injection. **F** Extreme limiting dilution analysis in vivo. **G** RT-qPCR analysis of EpCAM in shCtrl cells-derived tumor tissues and shCD146 cells-derived tumor tissues from mice. **H** IHC analysis of the proliferation marker Ki-67 in PLC/PRF/5-shCtrl cells-derived tumor tissues and PLC/PRF/5-shCD146 cells-derived tumor tissues from mice. Representative images (Left), Scale bars = 100 μm and quantification of expression of Ki-67 in three regions of an image (Right) were shown. Data are representative of at least three independent experiments and shown as mean ± s.d. (*p < 0.05; **p < 0.01; ***p < 0.001).

Next, we examined whether the knockdown of CD146 decreased cancer stemness in HCC. Because of the high expression of CD146 in PLC/PRF/5 and Huh7 cells (Fig. S1), we knocked down CD146 in PLC/PRF/5 (shCD146#1, shCD146#2) and Huh7 cells with lentivirus plasmids expressing CD146 shRNA and confirmed strong reduction of CD146 expression mRNA and protein levels (Fig. 3A). CD146 knockdown dramatically decreased the expression of Oct-4, EpCAM, and Nanog mRNA levels compared with controls (Fig. 3B). CD146 knockdown resulted in markedly decreased numbers of tumor spheres in PLC/PRF/5 and Huh7 cells, indicating CD146 knockdown suppressed tumor sphere formation (Fig. 3C). Meanwhile, the ELDA showed that the sphere formation efficiency of shCD146 cells was weaker than that of control cells in PLC/PRF/5 cells (Fig. S2B). This further indicated that CD146 plays a vital role in regulating the self-renewal ability of liver CSCs. Additionally, IC_50_ was determined of cisplatin in PLC/PRF/5 cells, which the measured value was 7.4μg/ml (Fig. S3). Resistance to cisplatin experiments were performed with reference to the working concentration determined by the IC_50_. CD146 knockdown caused cells to be more sensitive to cisplatincompared with control cells (Fig. 3D).

To further verify the role of CD146 in tumor initiation, the xenograft mouse model was generated by the injection of CD146 knockdown PLC/PRF/5 cells (shCD146#2) and control cells in NOD-SCID mice. Limiting dilution assays in vivo demonstrated that, when an identical number of tumor cells was injected, mice inoculated with PLC/PRF/5-shCD146#2 developed less subcutaneous tumors compared to those in the PLC/PRF/5-shCtrl group. (Fig. 3E). Notably, mice injected with PLC/PRF/5-shCD146#2 showed a significant reduction in subcutaneous tumors at a concentration of 2 × 10^5^ (Fig. 3E). Utilizing the online analytical tool ELDA, we found that the proportion of stem cells in the PLC/PRF/5-shCD146#2 group was markedly reduced compared to the PLC/PRF/5-shCtrl group (Fig. 3F). Moreover, CD146 knockdown markedly reduced the mRNA expression of the stemness gene EpCAM in the xenograft tumors (Fig. 3G). IHC analysis showed that the expression of the proliferation marker Ki-67 was suppressed (Fig. 3H). These results demonstrated that CD146 knockdown decreased the stemness and chemoresistance of HCC cells.

### CD146 activates Notch signaling pathways by upregulating JAG2 in HCC

**Fig. 4:**
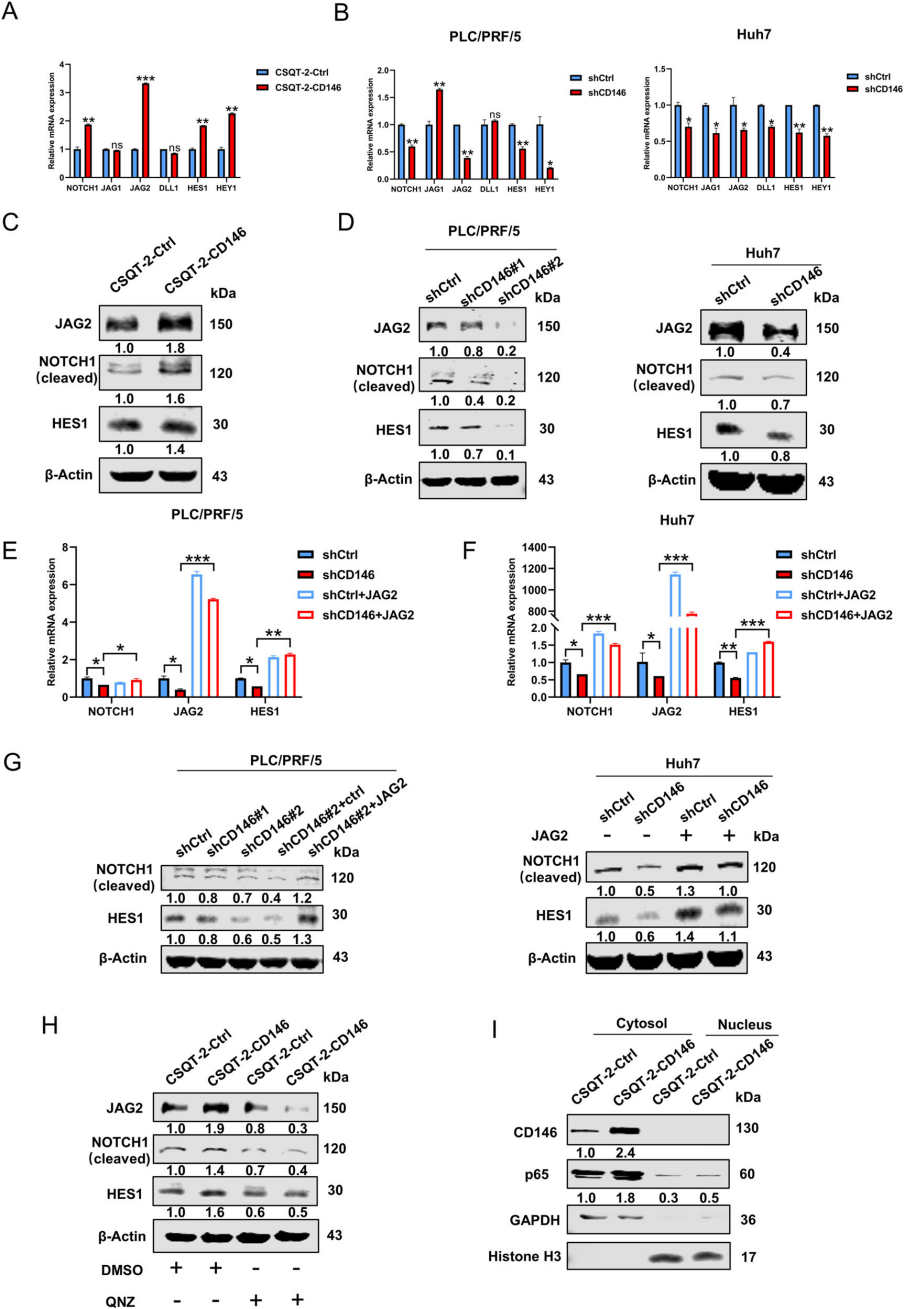
CD146 activates Notch signaling pathway via up-regulating JAG2 in HCC cells. **A**, **B** RT-qPCR analysis of Notch signaling pathway genes in CSQT-2-Ctrl and CSQT-2-CD146 cells (**A**) or shCtrl and shCD146 cells (**B**). The protein levels of JAG2, NOTCH1 and HES1 in CSQT-2-Ctrl and CSQT-2-CD146 cells (**C**) or shCtrl and shCD146 cells (**D**). **E**, **F** RT-qPCR analysis of JAG2, NOTCH1 and HES1 in shCtrl, shCD146 cells, shCtrl with stably JAG2 overexpression, shCD146 cells with stably JAG2 overexpression. **G** NOTCH1 and HES1 in shCtrl, shCD146 cells, shCtrl with stably JAG2 overexpression, shCD146 cells with stably JAG2 overexpression were detected by western blotting. **H** JAG2, NOTCH1 and HES1 in CSQT-2-Ctrl and CSQT-2-CD146 cells treated with DMSO or QNZ(EVP4593) (NF-κB signaling inhibitor, 5 μM) for 24 h were detected by western blotting. **I** Western blot analysis of p65 in the cytoplasm and nucleus of CSQT-2-Ctrl and CSQT-2-CD146 cells. Data are representative of at least three independent experiments and shown as mean ± s.d. (*p < 0.05; **p < 0.01; ***p < 0.001; ns, not significant).

The Hedgehog, Wnt, and Notch signaling pathways are involved in the regulation of cancer stemness [5, 10]. Notably, the Notch signaling pathway was the most strongly correlated with CD146 in the TCGA database (Fig. S4). We thus speculated that CD146 may regulate cancer stemness through the Notch signaling pathways in HCC. To prove this hypothesis, we detected the expression levels of ligands, receptors, and target genes in the Notch signaling pathway. In CSQT-2 cells, CD146 overexpression led to up-regulated mRNA expression levels of Notch signaling genes including JAG2, HES1, and HEY1; JAG2 was most significantly up-regulated gene (Fig. 4A). In contrast, CD146 knockdown decreased the mRNA expression levels of the Notch signaling genes in PLC/PRF/5 and Huh7 cell lines (Fig. 4B). Meanwhile, CD146 overexpression also up-regulated the protein expression levels of Notch signaling factors including NOTCH1, JAG2, and HES1 (Fig. 4C). Inversely, CD146 knockdown also decreased the protein level of NOTCH1, JAG2, and HES1, and the effect was most pronounced in PLC/PRF/5-shCD146#2.(Fig. 4D). CSQT-2-Ctrl cells, CSQT-2-CD146 cells, PLC/PRF/5-shCtrl and PLC/PRF/5-shCD146 cells were injected subcutaneously into the right flank of male NOD-SCID mice. After one month, these tumors were harvested for in vivo studies. we observed that CD146 overexpression enhanced the transcription of JAG2 and HES1 (Fig. S5A). In contrast, CD146 knockdown decreased the mRNA level of JAG2 and HES1 (Fig. S5B). CD146 overexpression only increased the protein expression of NOTCH1 and HES1 (Fig. S5C). Analogously, IHC analysis revealed that the expression of JAG2 was elevated in CD146-overexpressing CSQT-2 cells compared with CSQT-2 cells (Fig. S5D). Additionally, CD146 knockdown down-regulated the protein level of NOTCH1 and HES1 (Fig. S5E). IHC analysis showed that the expression of JAG2 was reduced in shCD146 PLC/PRF/5 cells (Fig. S5F). These results demonstrated that CD146 probably activates Notch signaling pathway by upregulating JAG2 in HCC.

To further verify that JAG2 plays a key role in CD146 regulation of Notch signaling pathway. We next stably overexpressed JAG2 in CD146 knockdown PLC/PRF/5 and Huh7 cells with lentiviral vectors, and the mRNA and protein expression levels of NOTCH1 and HES1 were examined. The mRNA and protein expression levels of NOTCH1 and HES1 in shCD146 cells were up-regulated after JAG2 overexpression (Fig. 4E–G). These results indicated that JAG2 overexpression in shCD146 cells restored Notch signaling pathway activity. Notably, it was found that overexpression of JAG2 increased the expression of CD146 (Fig. S6A-B). This finding suggested that JAG2 may positively regulate CD146.

A previous study showed that Jagged1 (JAG1) was up-regulated by endogenous NF-κB activation [35]. CD146 was previously shown to activate the NF-κB pathway [36, 37]. We thus speculated that CD146 may up-regulate JAG2 through activating NF-κB signaling. Therefore, CD146-overexpressing cell lines were treated with NF-κB signaling inhibitor QNZ-EVP4593. The protein level of JAG2 was examined. After treatment with QNZ-EVP4593, the upregulation of JAG2, NOTCH1, and HES1 caused by CD146 overexpression was antagonized (Fig. 4H), which indicated CD146 up-regulated JAG2 through NF-κB signaling activation. Together, these results suggested that CD146 likely up-regulated JAG2 expression and activated the Notch signaling pathway through NF-κB pathway in HCC cells. NF-κB is a protein complex that serves as a critical nuclear transcription factor within cells. The transcriptional activity of NF-κB is modulated by the phosphorylation or acetylation of its p65 subunit. Consequently, we assessed both the expression levels and nuclear localization of p65. We found that CD146 overexpression caused p65 to translocated to the nucleus by western blot analysis of nuclear and cytoplasm fractions (Fig. 4I). It also indicated that CD146 could activate NF-κB pathway. Unfortunately, how it activates JAG2 remains to be elucidated.

### CD146 regulation of HCC stemness depends on the Notch signaling pathway

**Fig. 5:**
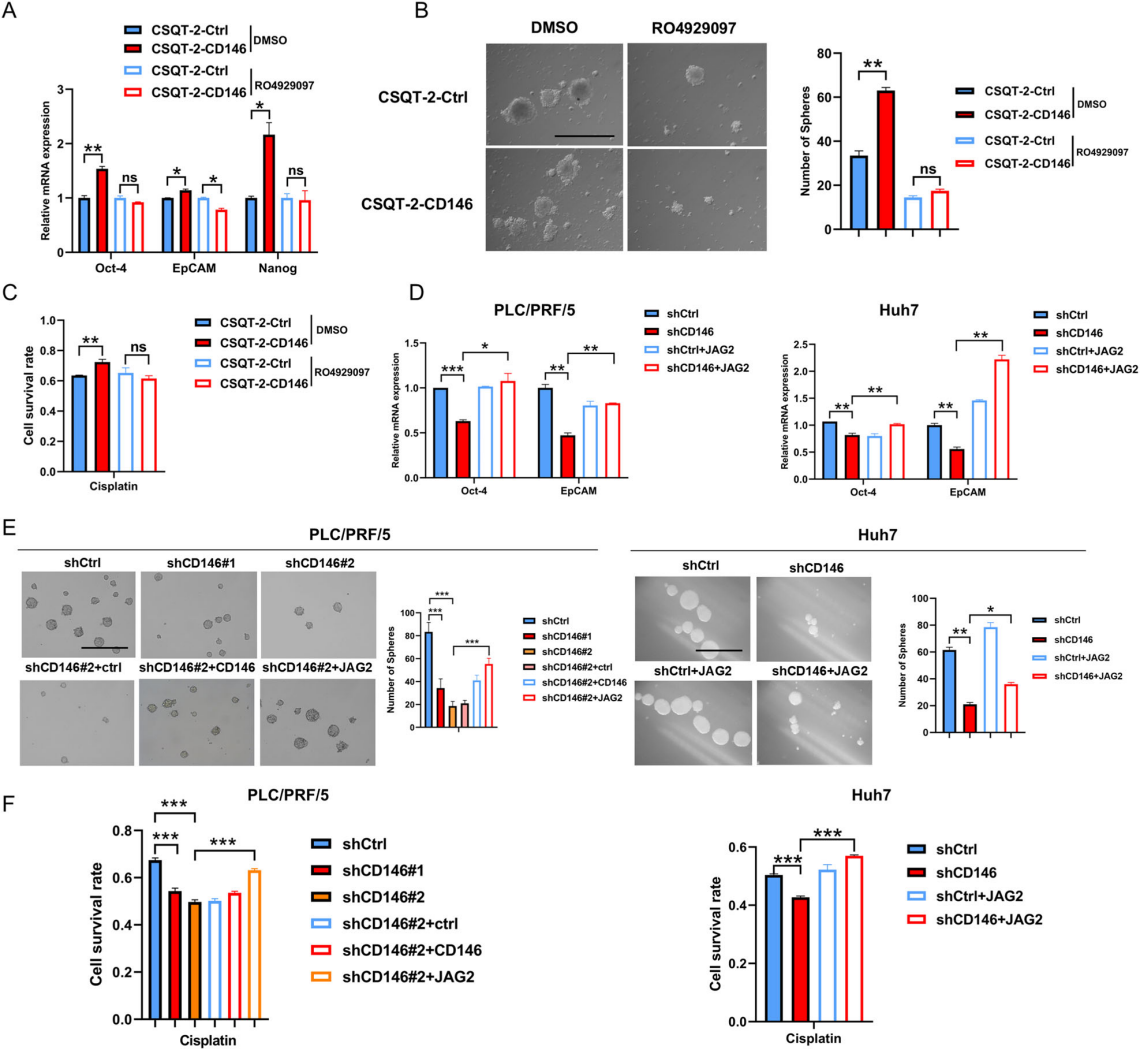
CD146 regulates HCC stemness depends on the Notch signaling pathway. **A** RT-qPCR analysis of Oct-4, EpCAM, Nanog were carried out in CSQT-2-Ctrl and CSQT-2-CD146 cells treated with DMSO or RO4929097 (Notch signaling pathway inhibitor, 10 μM) for 24 h. **B** Sphere formation ability of CSQT-2-Ctrl and CSQT-2-CD146 cells treated with DMSO or RO4929097 (10 μM) for 24 h. Scale bars = 120 μm. **C** CSQT-2-Ctrl and CSQT-2-CD146 cells were treated with DMSO or RO4929097 (10 μM) for 24 h, following were treated with indicated concentrations of cisplatin for 48 h. CCK-8 assay was used to measure cell viability. **D** RT-qPCR analysis of Oct-4, EpCAM were executed in shCtrl, shCD146, shCtrl with stably JAG2 overexpression, shCD146 cells with stably JAG2 overexpression. **E** Comparison of sphere formation ability between shCtrl, shCD146, shCtrl with stably JAG2-overexpressing, shCD146 cells with stably JAG2 overexpression by accessing number of tumor spheres (7 days). Scale bars = 120 μm. **F** The cell lines of shCtrl, shCD146#1, shCD146#2, shCD146#2 with control virus, shCD146#2 with stably CD146 overexpression, shCD146#2 with stably JAG2 overexpression PLC/PRF/5 cells were exposed to cisplatin at the concentration corresponding to its IC50 value for 48 h. The cell lines of shCtrl, shCD146, shCtrl with stably JAG2 overexpression, shCD146 with stably JAG2 overexpression Huh7 cells were treated with indicated concentrations of cisplatin for 48 h. CCK-8 assay was used to measure cell viability. Data are representative of at least three independent experiments and shown as mean ± s.d. (*p < 0.05; **p < 0.01; ***p < 0.001; ns not significant).

Next, the role of Notch signaling pathway in the effect of CD146 on HCC stemness was investigated. In CD146-overexpressing cells, the Notch signaling pathway inhibitor RO4929097 was used and the cancer cell stemness was examined. Upon treatment with RO4929097, the upregulation of stemness-related genes such as Oct-4, EpCAM, and Nanog induced by overexpression of CD146 was eliminated (Fig. 5A). Additionally, upon treatment with the inhibitor, the sphere formation ability of CSQT-2-CD146 cells declined (Fig. 5B). These results indicated that CD146 promoted the self-renewal capability of liver CSCs depends on the Notch signaling pathway. Upon treatment with RO4929097, CSQT-2-CD146 cells were more sensitive to cisplatin (Fig. 5C). Moreover, after treatment with the NF-κB signaling inhibitor QNZ-EVP4593, cells with overexpression of CD146 did not show elevation of stemness-related genes, sphere formation ability (Fig. S7A, B).

Conversely, JAG2 overexpression restored the transcription of stemness marker genes in CD146 knockdown HCC cells (Huh7 and PLC/PRF/5 cells) (Fig. 5D). JAG2 overexpression also increased the sphere numbers of shCD146 cells, indicating JAG2 overexpression rescued the sphere formation ability of shCD146#2 cells (Fig. 5E). Meanwhile, the ELDA showed that JAG2 overexpression enhanced the sphere formation efficiency of shCD146#2 cells (Fig. S8) Furthermore, exogenous expressed JAG2 reversed the reduction of chemoresistance caused by knockdown of CD146 (Fig. 5F). Together, these data indicated that CD146 regulates HCC stemness via Notch signaling pathway.

### CD146 is associated with the poor prognosis of HCC

**Fig. 6:**
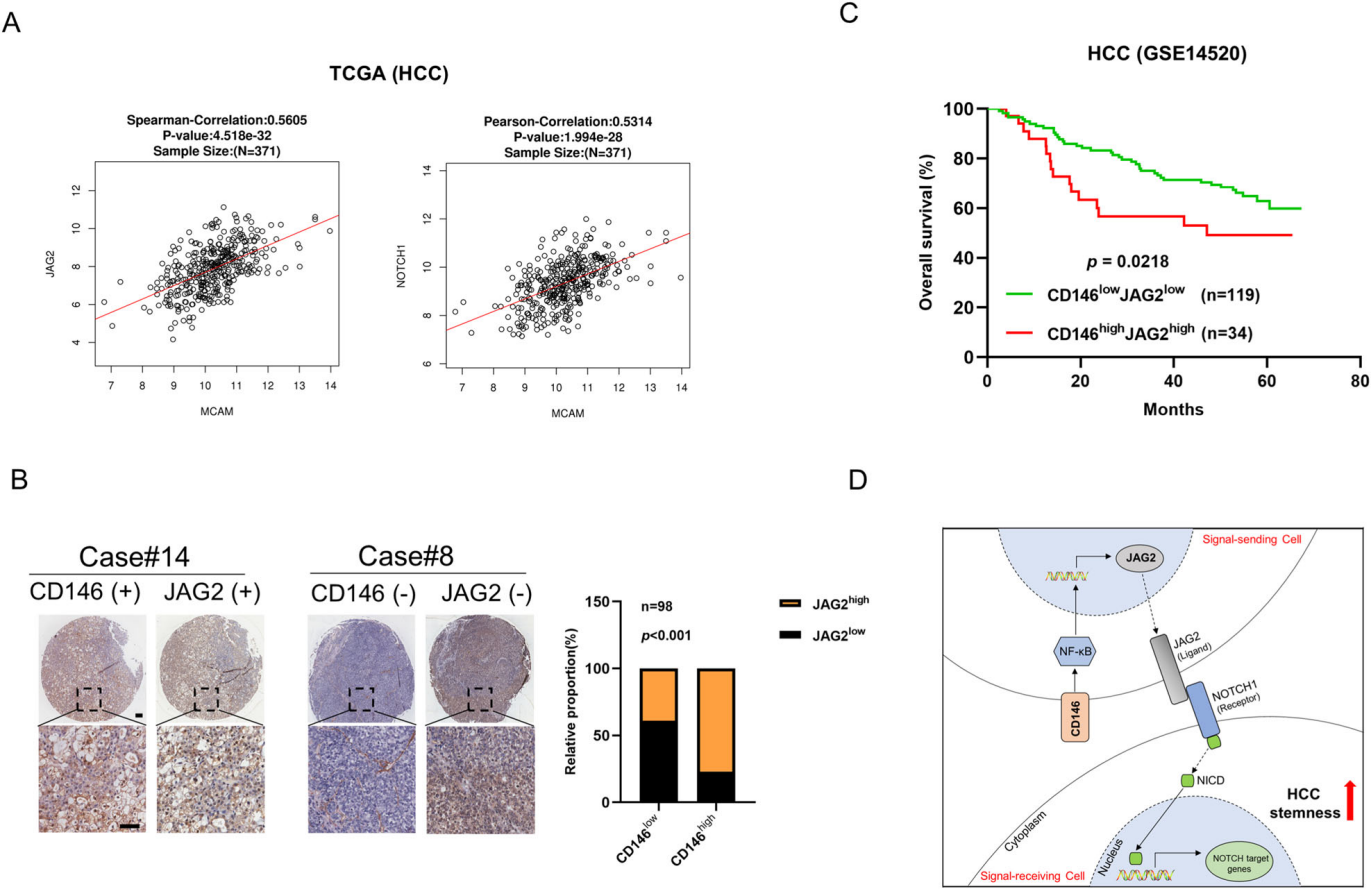
CD146 is associated with Notch signaling pathway and affects prognosis in HCC patients. **A** Correlation analysis of CD146 and the key genes of Notch signaling pathway (JAG2 and NOTCH1) in the TCGA database (HCC). **B** Left: IHC analysis of CD146 and JAG2 expressions in human HCC samples. Scale bars = 100 μm. Right: Correlation analysis of CD146 and JAG2 in human HCC samples. **C** Kaplan-Meier survival curve of overall survival (OS) for patients with CD146^high^JAG2^high^ and CD146^low^JAG2^low^ in HCC patients from the GSE database (GSE14520). **D** The signaling pathway pattern diagram for CD146 regulates HCC stemness.

Analysis of TCGA revealed that Notch signaling pathway genes including JAG2 and Notch1 were positively correlated with CD146 (Fig. 6A). IHC showed that CD146 expression was positively correlated with JAG2 in HCC tissue samples (Fig. 6B). These results revealed that CD146 is positively correlated with the Notch signaling pathway in HCC. The clinical management of cancer patients should pay more attention to the survival rate. In the analysis of dataset GSE14520, patients with CD146^low^JAG2^low^ showed a better overall survival compared with patients with CD146^high^JAG2^high^ (Fig. 6C). Compared with patients with CD146^low^JAG2^low^, patients with CD146^high^JAG2^high^ exhibited worse disease-free survival (Fig. S9A) and overall survival (Fig. S9B) in the TCGA database. Taken together, these data indicated that CD146 is positively associated with the Notch signaling pathway and affects prognosis in HCC patients. Moreover, low expression of both CD146 and JAG2 is more favorable for survival in HCC patients. These results suggested the potential of targeting both CD146 and JAG2 as a therapeutic strategy for HCC.

## Discussion

Despite advances in the treatment of HCC, the long-term survival of patients with HCC is still unsatisfactory. CD146, also known as MUC18 or MCAM, was first identified in malignant melanoma and plays an important role in driving melanoma progression and metastasis [16]. CD146 plays a vital role in the oncogenesis and progression of many cancers, and CD146 expression have been shown to be elevated in prostate, epithelial ovarian and breast cancer [38–40]. CD146 expression was found to be up-regulated in HCC tissues and stimulated tumorigenesis [26, 41]. Previous studies have also shown that CD146 is a novel marker for CSCs in HCC [41]. Analysis of TCGA database revealed that stemness-related genes were positively correlated with CD146 and differentiation-related genes were negatively correlated with CD146 in HCC. We then measured the expression of CD146 and stemness-related genes (such as CD133 and Oct-4) in HCC tissue samples and found that CD146 expression was positively correlated with Oct-4 and CD133. CD146 mRNA and protein expression were dramatically increased in spheres derived from HCC cells compared with the corresponding adherent cells. These findings suggested that CD146 was highly expressed in liver CSCs.

Sphere formation assays showed that overexpression of CD146 enhanced the self-renewal capacity of liver CSCs. Inversely, CD146 knockdown reduced the self-renewal capacity of liver CSCs. The overexpression of CD146 upregulated the transcription of Oct-4, EpCAM, and Nanog. Moreover, it was further confirmed that overexpression of CD146 enhanced tumorigenicity in xenograft models. Above results suggest that CD146 actively regulates cancer stemness in HCC. In line with our results, other studies showed that CD146 plays a crucial role in promoting the tumor growth of breast cancer cells and enhances the stemness of breast cancer cells [40]. Thus, the identification of signaling pathways involved in CD146-mediated cancer stemness regulation is important for the understanding of liver CSC biology and the development of novel anti-cancer therapies. Moreover, CD146 has been considered a potential tumor therapeutic target [42, 43].

Several signaling pathways are involved in the maintenance of CSC phenotypes, including Notch, Hedgehog, Wnt signaling pathways [44–47] Studies showed that Hippo [48, 49] signaling pathways also regulate the stemness of cancer cells in HCC. However, to the best of our knowledge, no studies have demonstrated that CD146 could regulate the stemness of HCC cells through the Notch signaling pathway. In TCGA database of HCC, CD146 showed the strongest positive correlation with the Notch signaling pathway (Fig. S4). In HCC tissue samples, CD146 expression was positively correlated with JAG2, suggesting that CD146 might play a crucial role in the Notch signaling pathway. A recent study reported that the Notch signaling pathway was significantly activated in CD146+ cells in primary human sarcoma [50]. In our study, overexpression of CD146 in CSQT-2 cells led to significant up-regulation of JAG2 (Fig. 4A). CD146 was knocked down in PLC/PRF/5 and Huh7 cells, and the majority of ligand and receptors of the Notch signaling pathway were down-regulated. JAG2 was the most significantly down-regulated protein examined. Similar results were obtained in vivo (Fig. S5). Thus, it was speculated that CD146 might activate the Notch signaling pathway through up-regulation of JAG2. To verify this possibility, we found that overexpression of JAG2 in shCD146 cells restored Notch signaling pathway activity. Notably, overexpression of JAG2 also increased the expression of CD146. This finding suggested that JAG2 may also positively regulate CD146. Such a positive feedback mechanism is conducive to tumor growth (Fig. S6).

A previous study indicated that Jagged1 (JAG1) can trigger the Notch signaling pathway via NF-κB signaling [35]. Both JAG1 and JAG2 are ligands in the Notch signaling pathway, and they up-regulate JAG1 expression to activate the Notch pathway. Numerous studies have shown that CD146 activates NF-κB signaling [36, 37] Thus, we speculated that CD146 might up-regulate the expression of JAG2 through the NF-κB signaling to activate the Notch signaling pathway. To test this conjecture, we used the NF-κB inhibitor QNZ-EVP4593 in CD146-overexpressing HCC cells, which reported the protein levels of JAG2, NOTCH1 and HES1 were down-regulated. These results indicate that CD146 up-regulates the expression of JAG2 through NF-κB signaling to activate the Notch signaling pathway. Even so, how CD146 up-regulates the expression of JAG2 remains unclear. As a member of the immunoglobulin-like adhesion molecule (Ig-CAM) superfamily, CD146 can mediate contraction between neighboring cells or between cells and extracellular matrix. Meanwhile, as a cell membrane receptor, CD146 is a cellular environmental receptor and signal transduction regulatory hub, mediating signal transduction. It is involved in the biological processes of cell adhesion, proliferation, differentiation, vascular and neural development, tumor development and metastasis [51]. G. Ahn et al. indicated that CD146 interacts with Wnt5a to activate the Wnt/PCP signaling pathway and promote melanoma metastasis [52]. A previous study indicated that CD146 interacts with VEGFR3 to activate the EPK pathway and promote the proliferation and migration of lymphatic endothelial cells [53]. Based on the above, we speculated that CD146 probably activates the JAG2-NOTCH pathway by activating the NF-κB signaling pathway. So, further studies are necessary to investigate the direct interaction between CD146 and JAG2 and elucidate the mechanism of HCC occurrence and development.

To verify whether CD146 regulates the stemness of HCC via the Notch signaling pathway, we used the Notch signaling pathway inhibitor RO4929097 in CD146-overexpressing cells and found that the self-renewal ability and chemoresistance of HCC cells were suppressed. When we overexpressed JAG2 in shCD146 cells, the self-renewal ability and chemoresistance were impaired. In CD146-overexpressing HCC cells, NF-κB inhibitor reduced the self-renewal ability (Fig. S7).

Numerous previous studies have shown that CD146 is associated with prognosis in patients with various types of cancer, including breast cancer [54–56], osteosarcoma [57], melanoma [58], and HCC [25]. In our study, through analysis of the GSE and TCGA databases, patients with CD146^low^JAG2^low^ showed better overall survival compared with patients with CD146^high^JAG2^high^. However, there were relatively few patients with CD146^high^JAG2^high^ in TCGA database, which inevitably led to statistical biases (Fig. S9). These results indicated that CD146/NF-κB/JAG2 signaling plays a vital role in regulating the stemness of HCC cells and was associated with HCC patient outcomes. Meaning, low expression of both CD146 and JAG2 is more favorable for survival in HCC patients. These results suggest the potential of targeting both CD146 and JAG2 as a therapeutic strategy for HCC. The mechanisms of CD146 regulating stemness of HCC cells by activating Notch signaling pathway are summarized in Fig. 6D.

In conclusion, our findings indicated that CD146/NF-κB/JAG2 signaling plays a positive role in regulating the stemness of HCC cells. Thus, targeting this signaling pathway might be a novel therapeutic strategy for the clinical management of HCC patients.

## Supplementary information

Supplementary Materials

Full length western blots

## Data Availability

All data needed to evaluate the conclusions in the paper are present in the paper and/or the Supplementary Materials. The data can be obtained in GEO under the accession number: GSE14520.
