# Supplementary material for: CD146 regulates the stemness and chemoresistance of hepatocellular carcinoma via JAG2-NOTCH signaling
Source: Cell Death Dis. 2025 Mar 3;16(1):150. doi: 10.1038/s41419-025-07470-x (PMC11876685; doi:10.1038/s41419-025-07470-x)

Supplementary Fig. 1 Western blot analysis of CD146 in HCC cells.

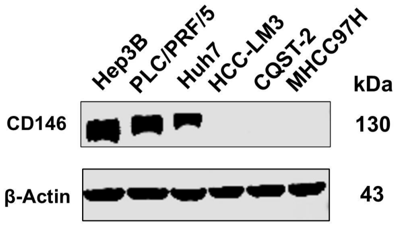

Supplementary Fig. 2 The Extreme limiting dilution analysis (ELDA) were performed to testing the viability of CD146 in CSQT-2-C， CSQT-2-CD146 (A), PLC/PRF/5-shCtrl, PLC/PRF/5-shCD146#1, PLC/PRF/5-shCD146#2 cells (B).

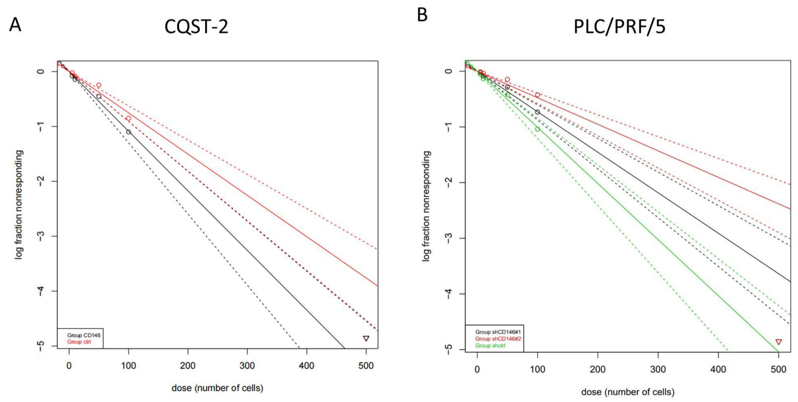

Supplementary Fig. 3 Half maximal inhibitory concentration (IC50) were determined of cisplatin in CSQT-2 and PLC/PRF/5 cells.

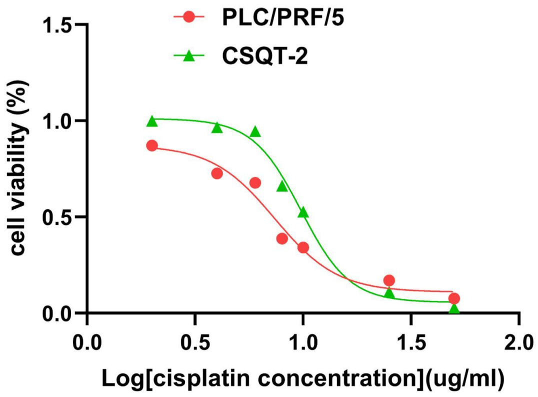

Supplementary Fig. 4 Correlation between CD146 and Notch signaling pathway in TCGA database (HCC).

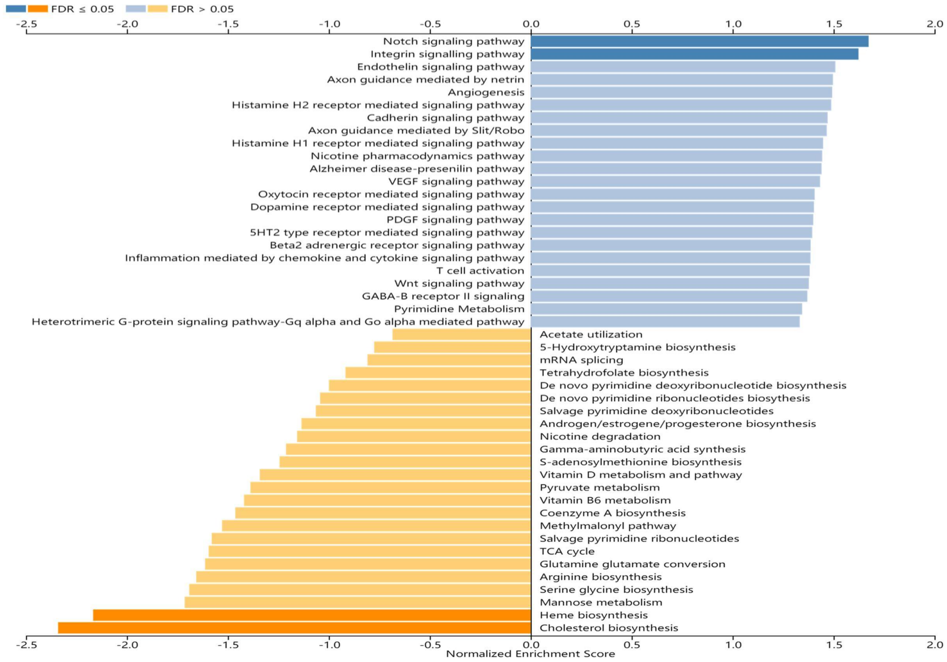

Supplementary Fig. 5 CD146 activates Notch signaling pathway in vivo. **A** RT-qPCR analysis of *CD146*, *JAG2* and *HES1* were executed in CSQT-2-CD146 cells-derived tumor tissues and CSQT-2-Ctrl cells-derived tumor tissues from mice. **B** RT-qPCR analysis of *CD146*, *JAG2* and *HES1* were executed in shCtrl cells-derived tumor tissues and shCD146 cells-derived tumor tissues from mice. **C** CD146, NOTCH1 and HES1 in CSQT-2-CD146 cells-derived tumor tissues and CSQT-2-Ctrl cells-derived tumor tissues from mice were detected by western blotting. **D** IHC analysis of JAG2 in CSQT-2-CD146 cells-derived tumor tissues and CSQT-2-Ctrl cells-derived tumor tissues from mice. Representative images (Left), Scale bars = 100 μm and quantification of expression of JAG2 in three regions of an image (Right) were shown. **E** CD146, NOTCH1 and HES1 in PLC/PRF/5-shCtrl cells-derived tumor tissues and PLC/PRF/5-shCD146 cells-derived tumor tissues from mice were detected by western blotting. **F** IHC analysis of JAG2 in PLC/PRF/5-shCtrl cells-derived tumor tissues and PLC/PRF/5-shCD146 cells-derived tumor tissues from mice. Representative images (Left), Scale bars = 100 μm and quantification of expression of JAG2 in three regions of an image (Right) were shown. Data are representative of at least three independent experiments and shown as mean ± s.d. (*p <0.05; ***p < 0.001;)

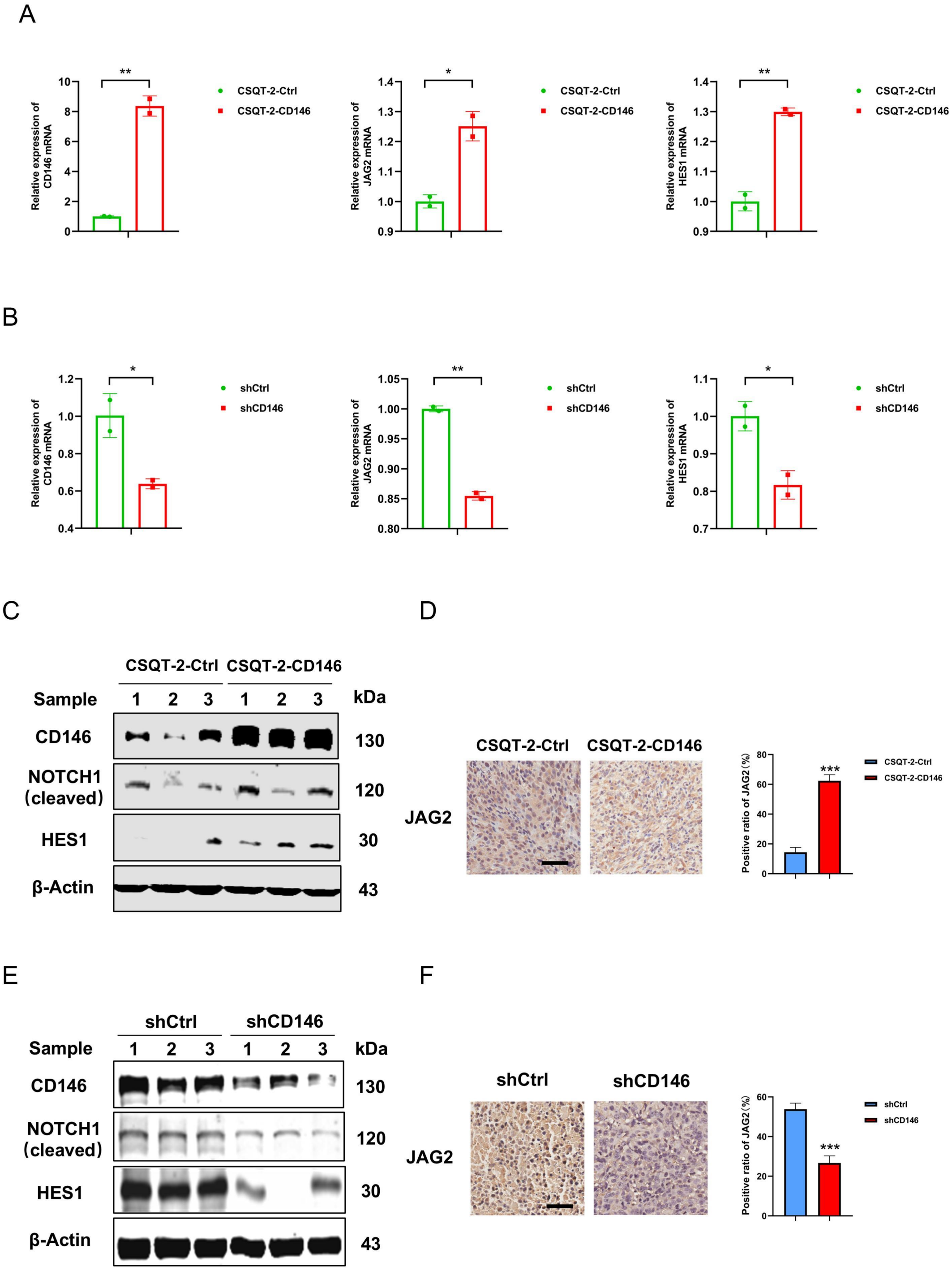
A
Relative expression of CD146 mRNA
Relative expression of JAG2 mRNA
Relative expression of HES1 mRNA
CSQT-2-Ctrl
CSQT-2-CD146
B
shCtrl
shCD146
C
CSQT-2-Ctrl
CSQT-2-CD146
Sample
kDa
CD146
NOTCH1 (cleaved)
HES1
β-Actin
130
120
30
43
D
JAG2
Positive ratio of JAG2(%)
E
shCtrl
shCD146
F
JAG2

Supplementary Fig. 6 JAG2 overexpression resulted in the upregulation of *CD146* in PLC/PRF/5 cells. **A** RT-qPCR analysis of *CD146* in shCtrl, shCD146, shCtrl with stably JAG2 overexpression, shCD146 cells with stably JAG2 overexpression. **B** The expression of CD146 in shCtrl, shCD146, shCtrl with stably JAG2 overexpression, shCD146 cells with stably JAG2 overexpression were measured by western blotting.

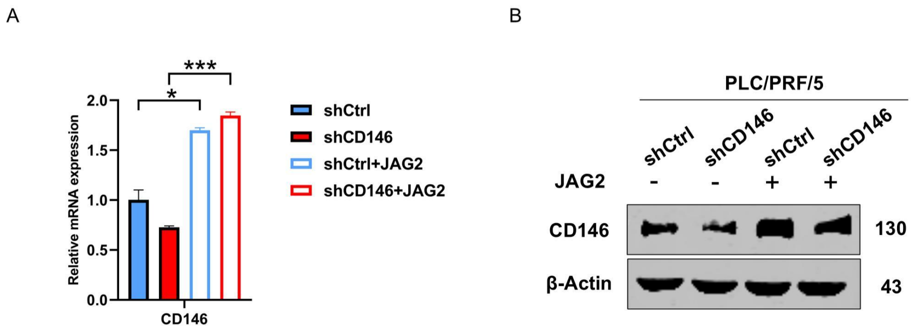

Supplementary Fig. 7 CD146 regulates stemness of HCC depends on activating NF-κB signaling. **A** RT-qPCR analysis of *Oct-4*, *EpCAM*, *Nanog* were carried out in CSQT-2-Ctrl and CSQT-2-CD146 cells treated with DMSO or QNZ(EVP4593) (NF-κB signaling inhibitor, 5μM) for 24h. **B** Sphere formation ability of CSQT-2-Ctrl and CSQT-2-CD146 cells treated with DMSO or QNZ(EVP4593) (5μM) for 24h. Scale bars=120μm. Data are representative of at least three independent experiments and shown as mean ± s.d. (*p <0.05; **p < 0.01; ***p < 0.001; ns not significant).

A

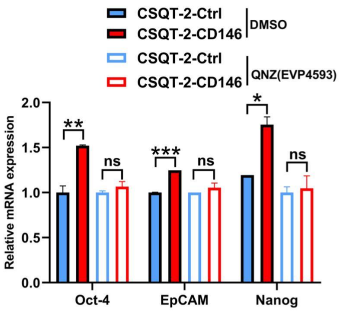

B

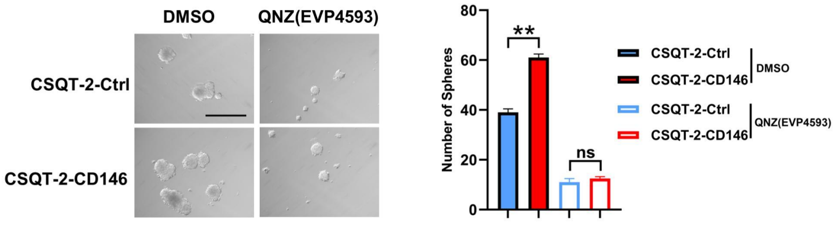

Supplementary Fig. 8 The Extreme limiting dilution analysis (ELDA) were performed to testing the viability of CD146 in shCtrl, shCD146#1, shCD146#2, shCD146#2 with control virus, shCD146#2 with stably CD146 overexpression, shCD146#2 with stably JAG overexpression PLC/PRF/5 cells.

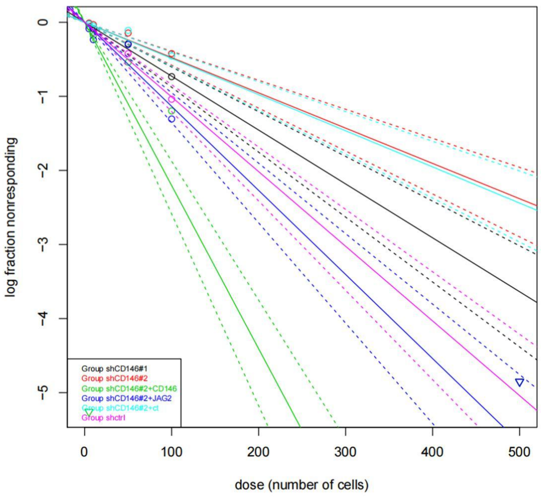

Supplementary Fig. 9 CD146/JAG2 affects prognosis of HCC patients. **A, B** Kaplan-Meier survival curve of disease-free (**A**) and overall survival survival (**B**) for patients with CD146^{high}JAG2^{high} and CD146^{low}JAG2^{low} in HCC patients from TCGA database. (*p <0.05)

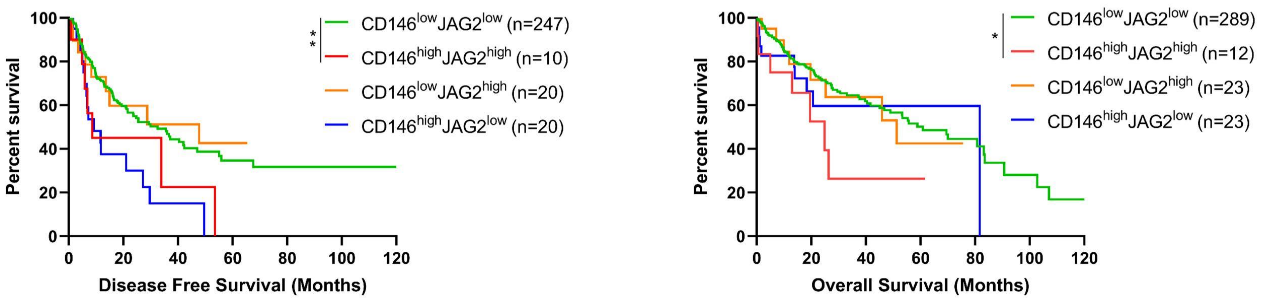

Supplement: Supplementary file 1 — Supplementary Materials [file 41419_2025_7470_MOESM1_ESM.pdf]
