# Supplementary material for: CD146 regulates the stemness and chemoresistance of hepatocellular carcinoma via JAG2-NOTCH signaling
Source: Cell Death Dis. 2025 Mar 3;16(1):150. doi: 10.1038/s41419-025-07470-x (PMC11876685; doi:10.1038/s41419-025-07470-x)
Supplement: Supplementary file 2 — Full length western blots [file 41419_2025_7470_MOESM2_ESM.pdf]

Full length western blots

Fig.1D

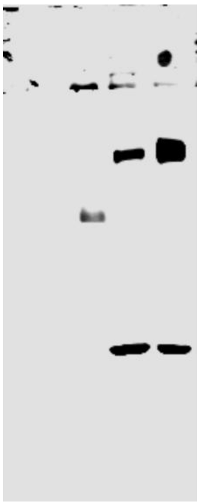

LM3+β-Actin

Fig.2A

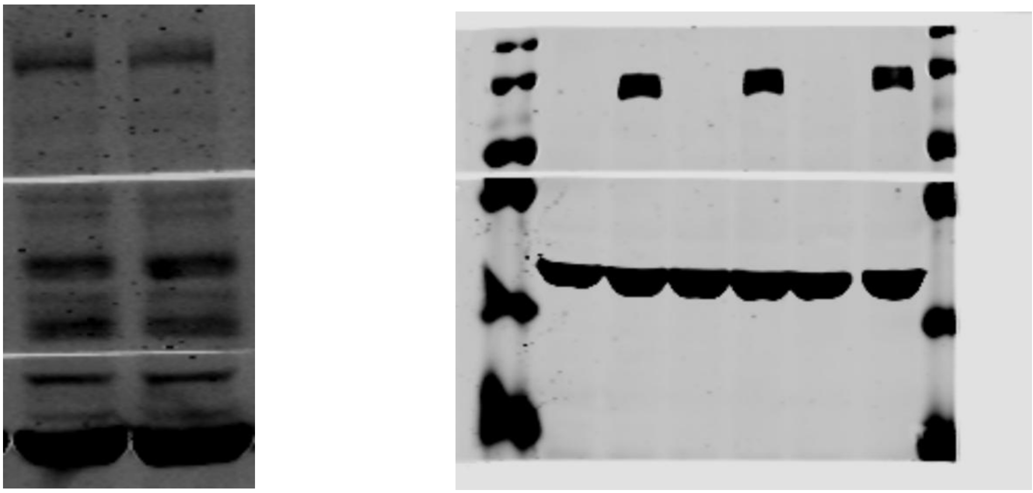

Huh7-CD146+β-Actin PLC-CD146+β-Actin

Fig.3A

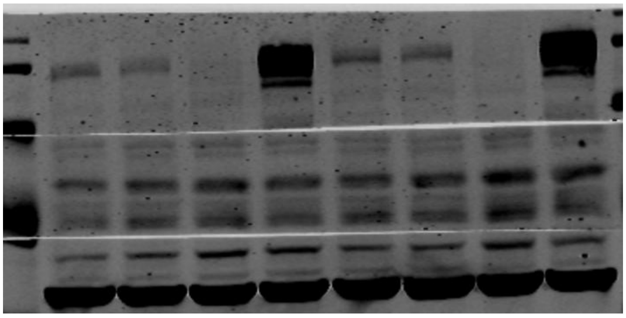

Huh7-CD146+β-Actin

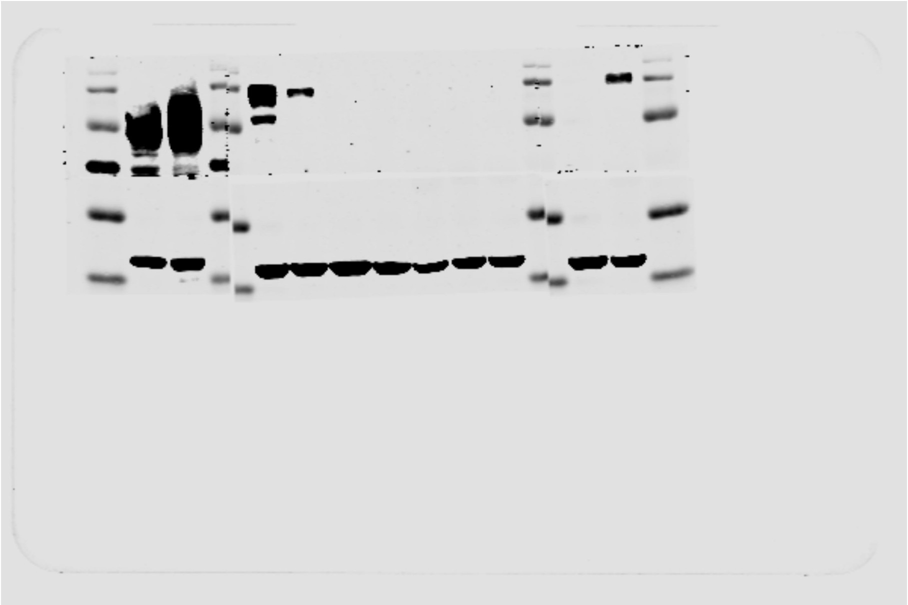

PLC-CD146+β-Actin

Fig.4C

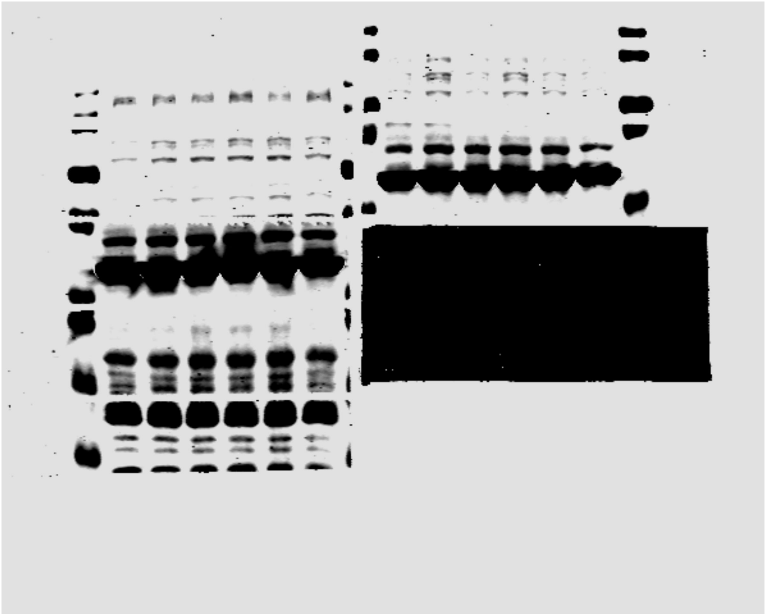

CSQT-2-HES1

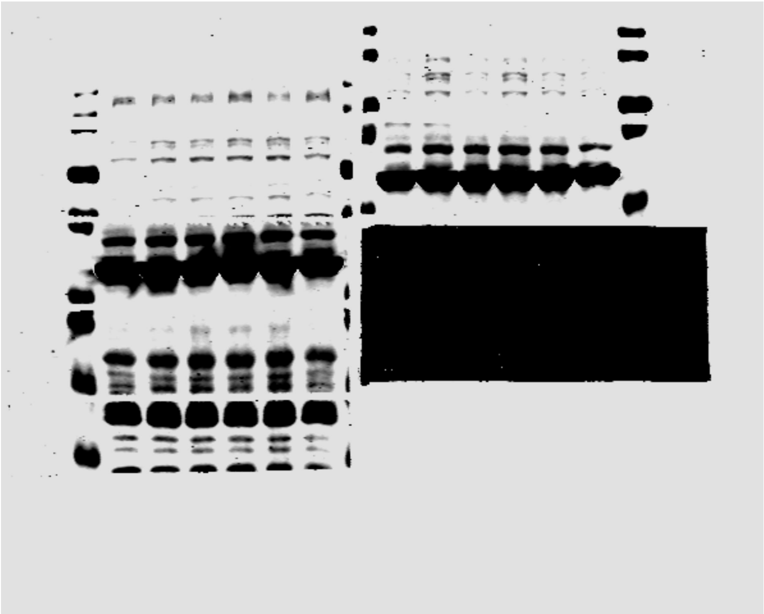

CSQT-2-JAG2-Notch1

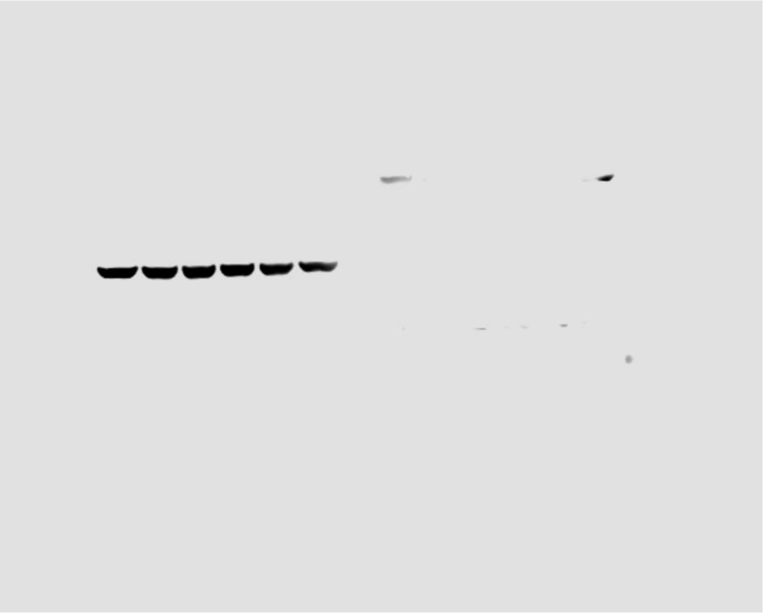

CSQT-2-β-Actin

Fig.4D

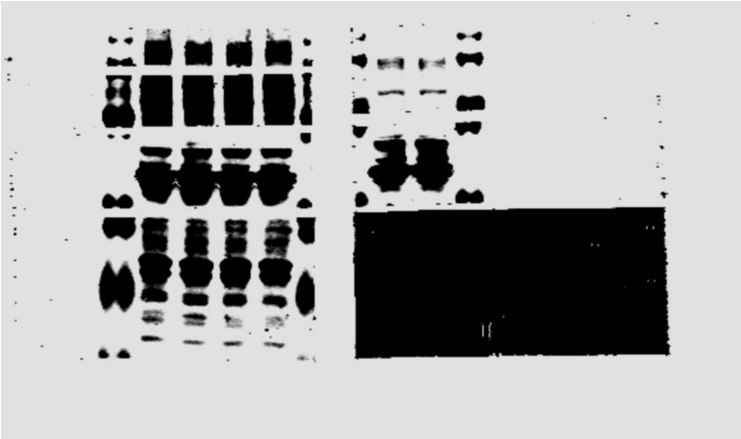

Huh7-HES1

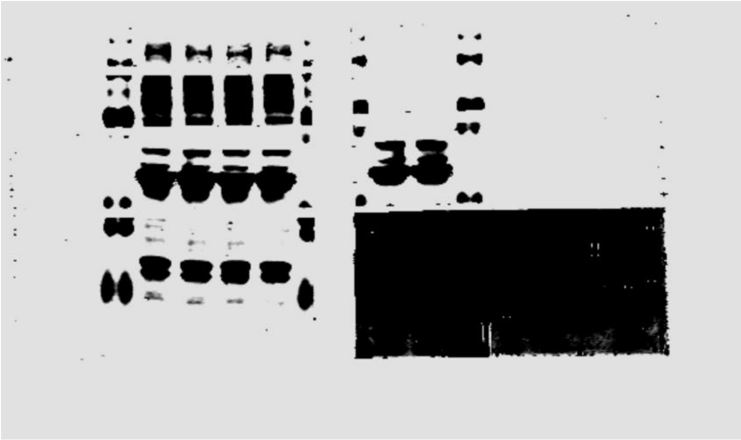

Huh7-JAG2

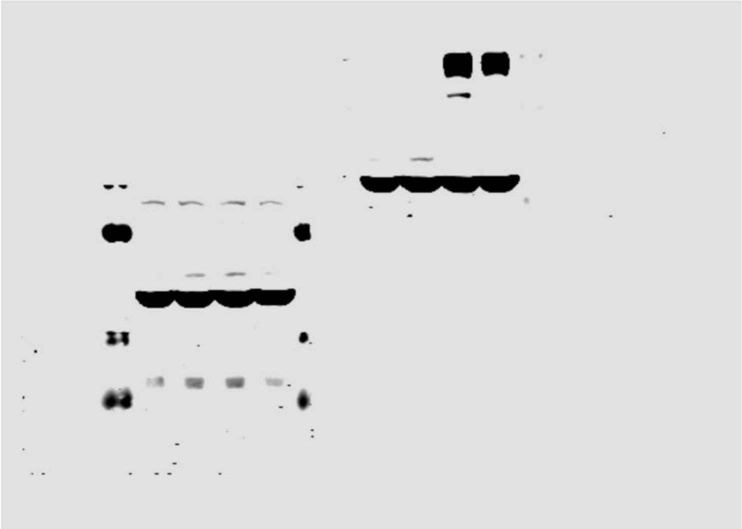

Huh7-Notch1

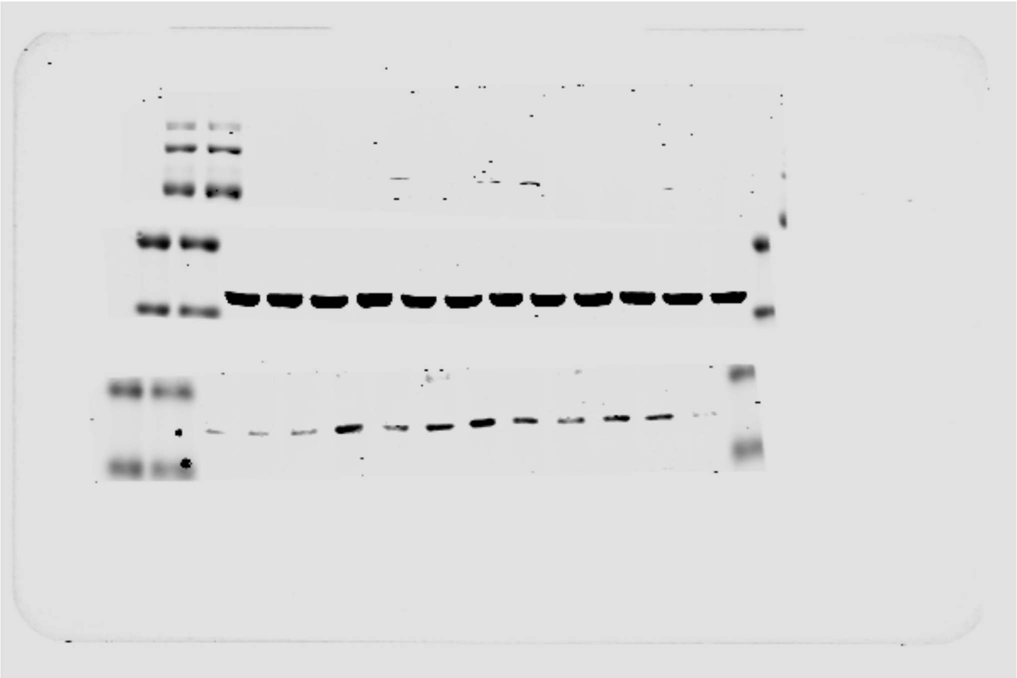

PLC-HES1

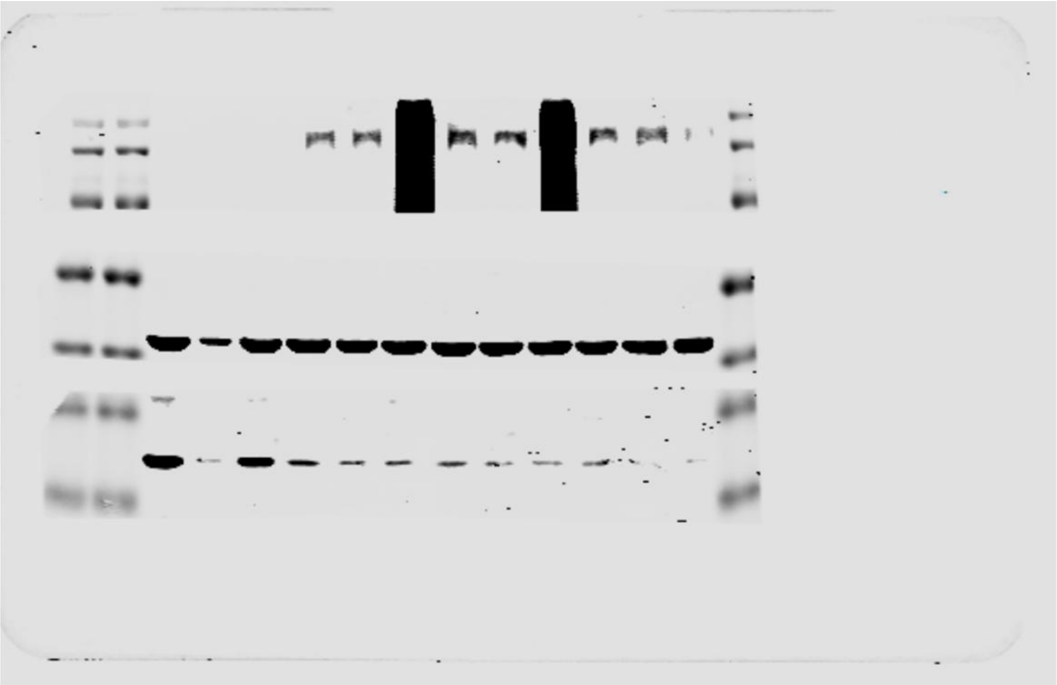

PLC-JAG2

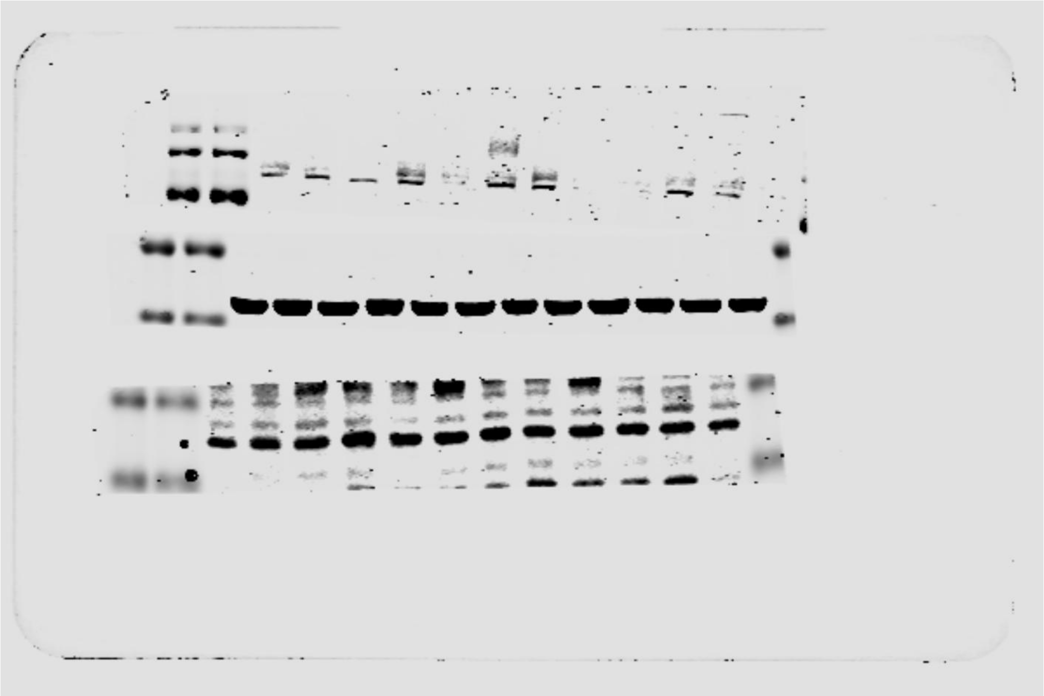

PLC-Notch1

Fig.4G

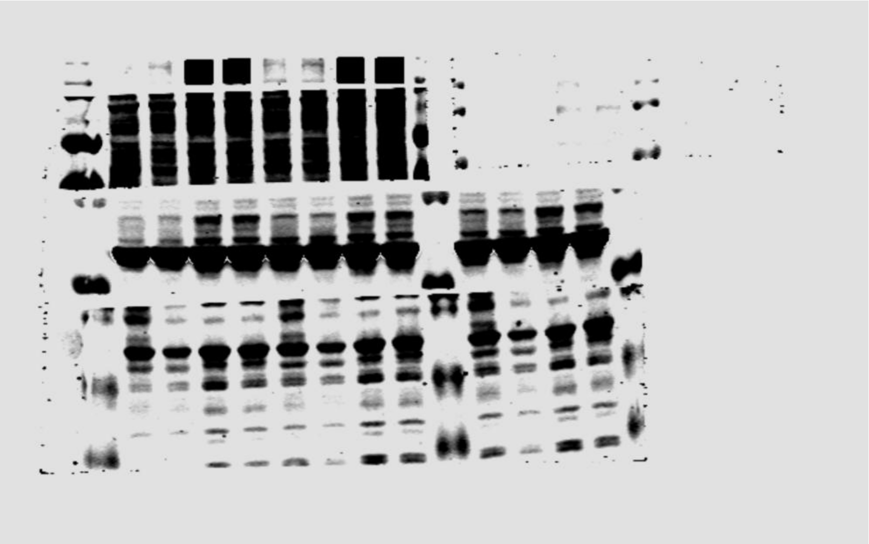

Huh7+JAG2-HES1

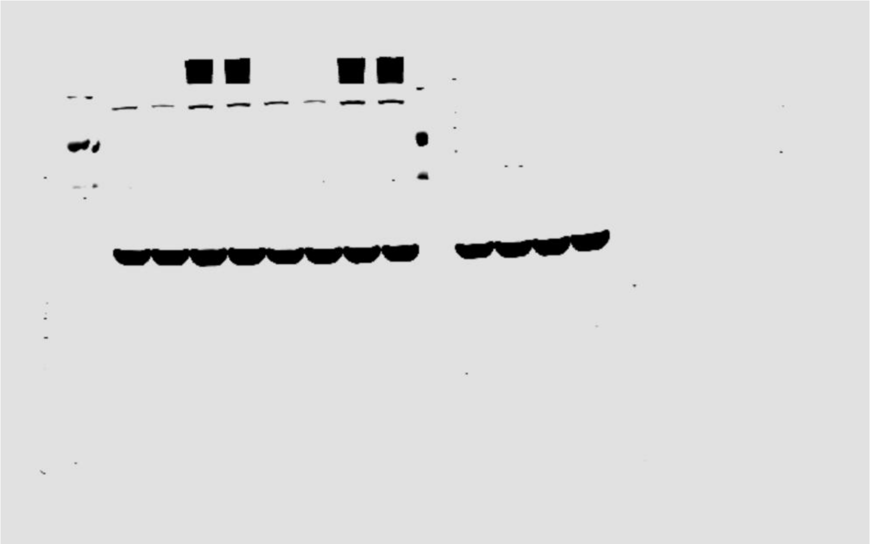

Huh7+JAG2-Notch1

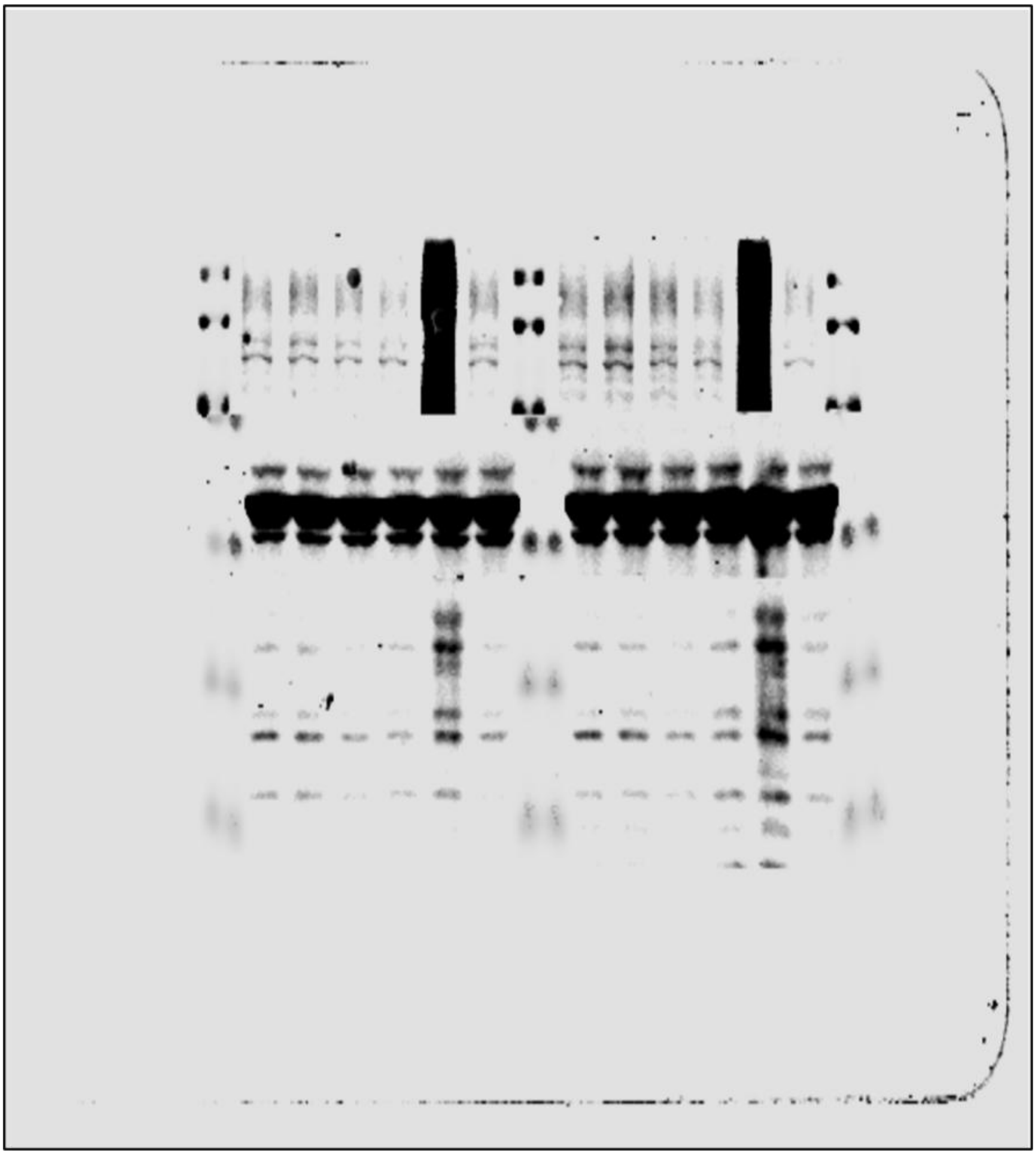

PLC+JAG2-HES1

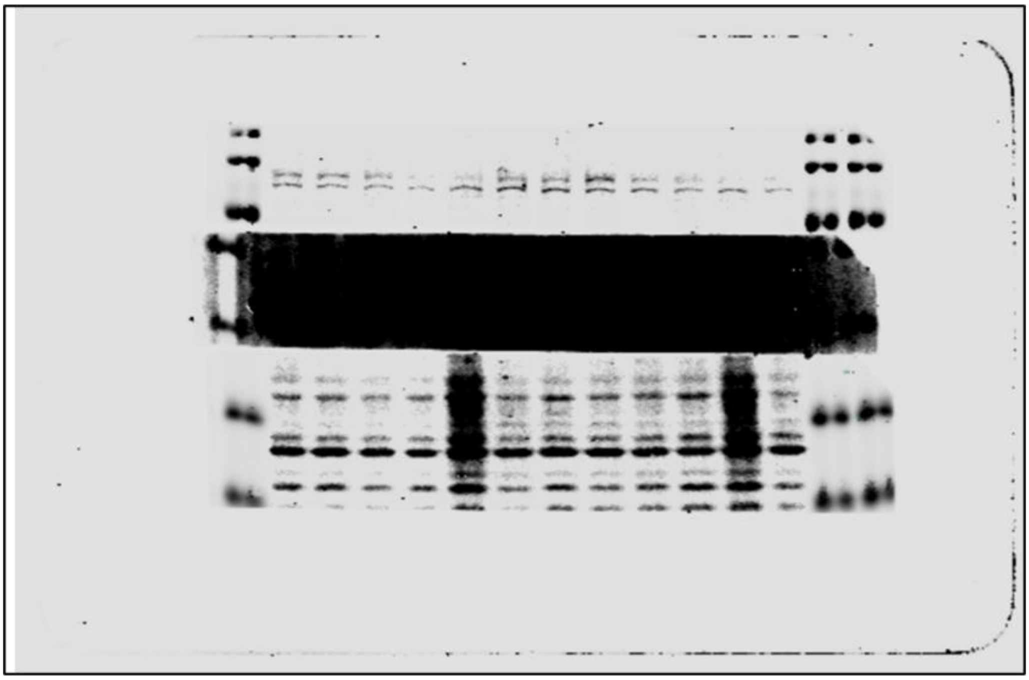

PLC+JAG2-NOTCH1

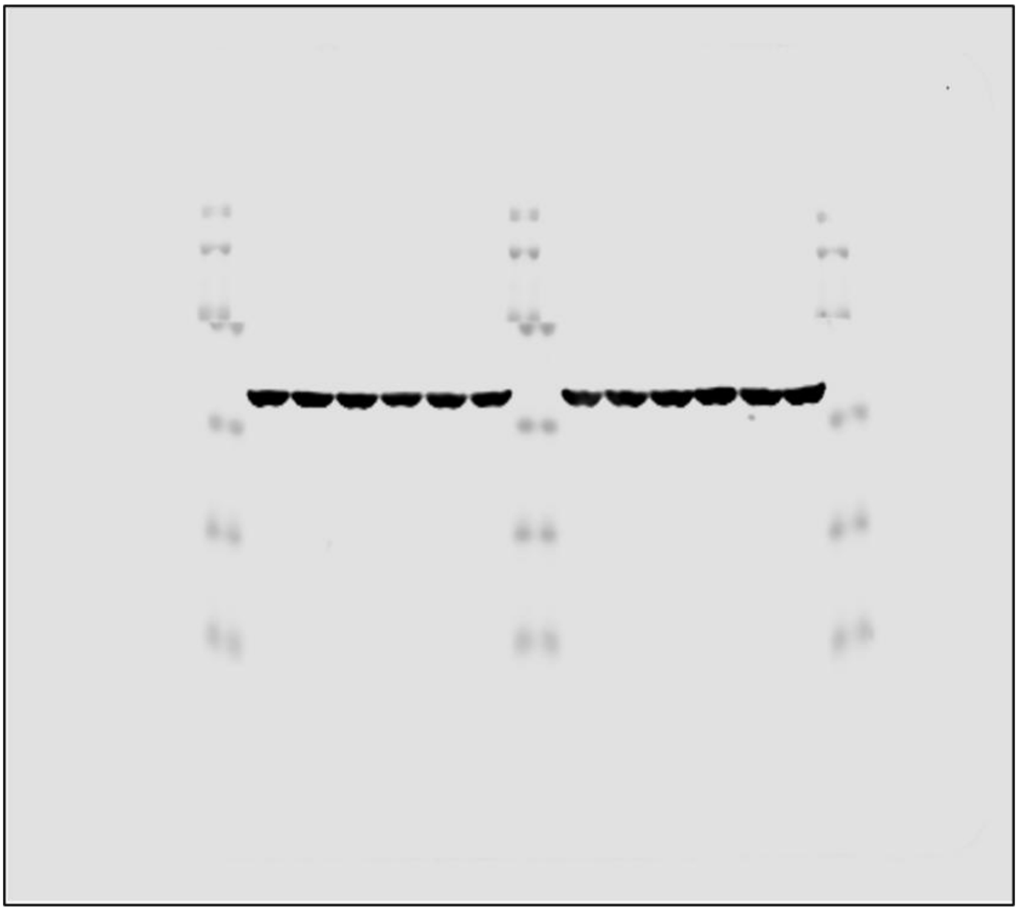

PLC β-Actin

Fig.4H

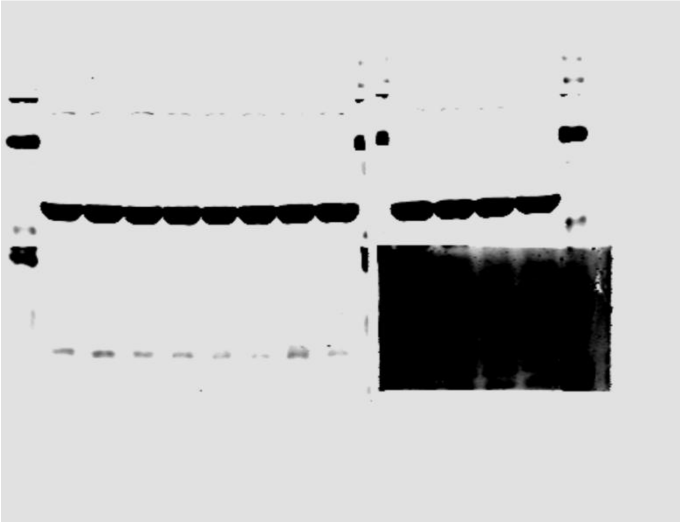

CSQT-2_QNZ-HES1

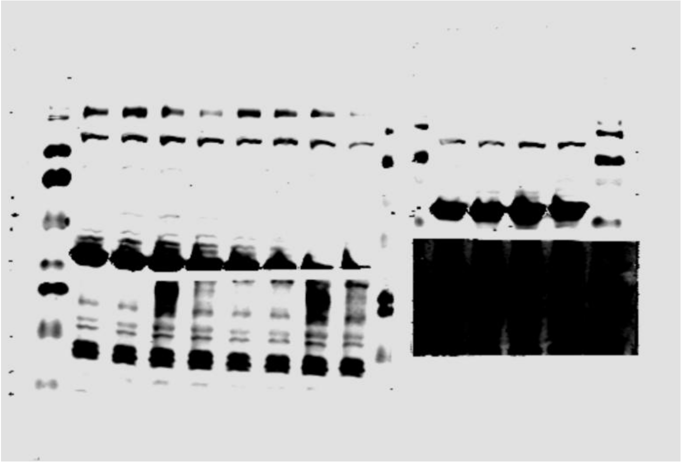

CSQT-2_QNZ-JAG2

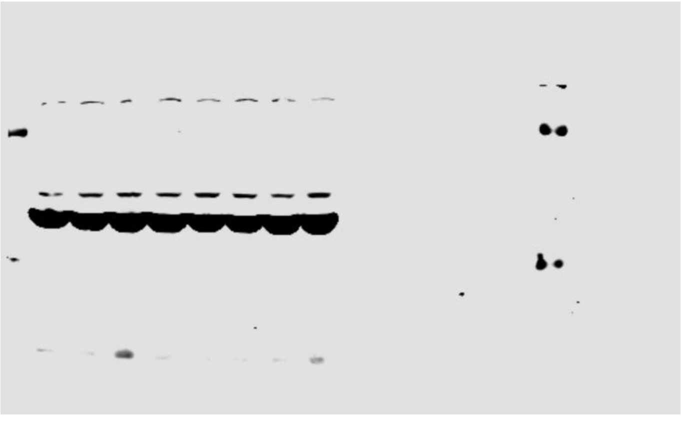

CSQT-2_QNZ-Notch1

Fig.4I

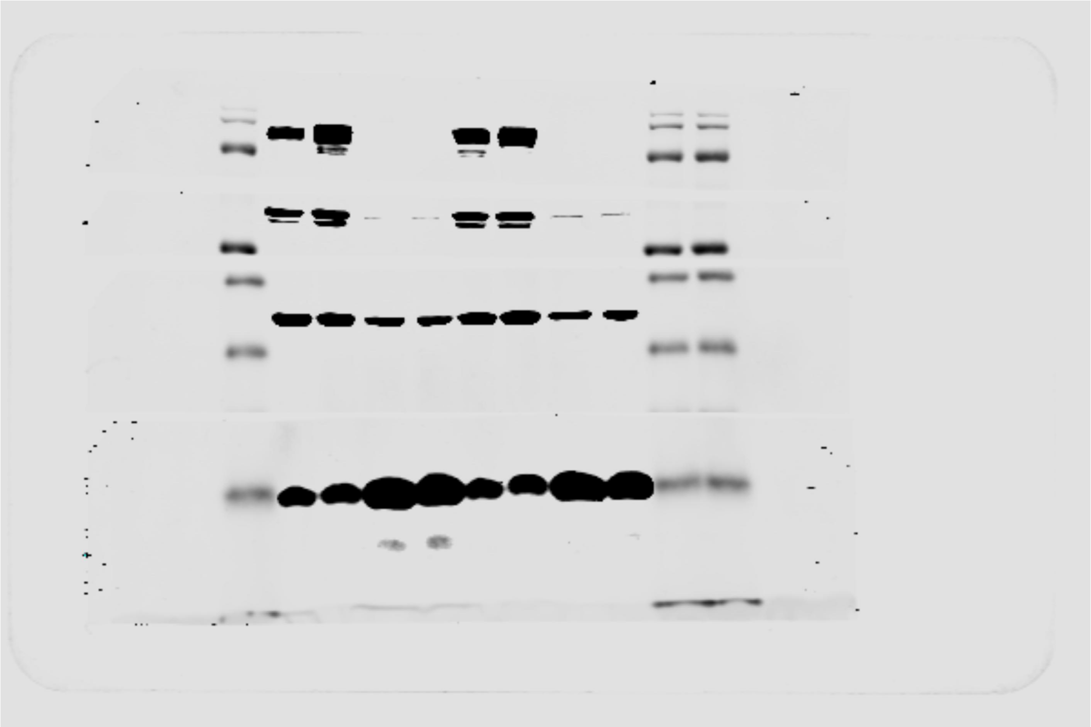

CSQT-2-CD146+P65

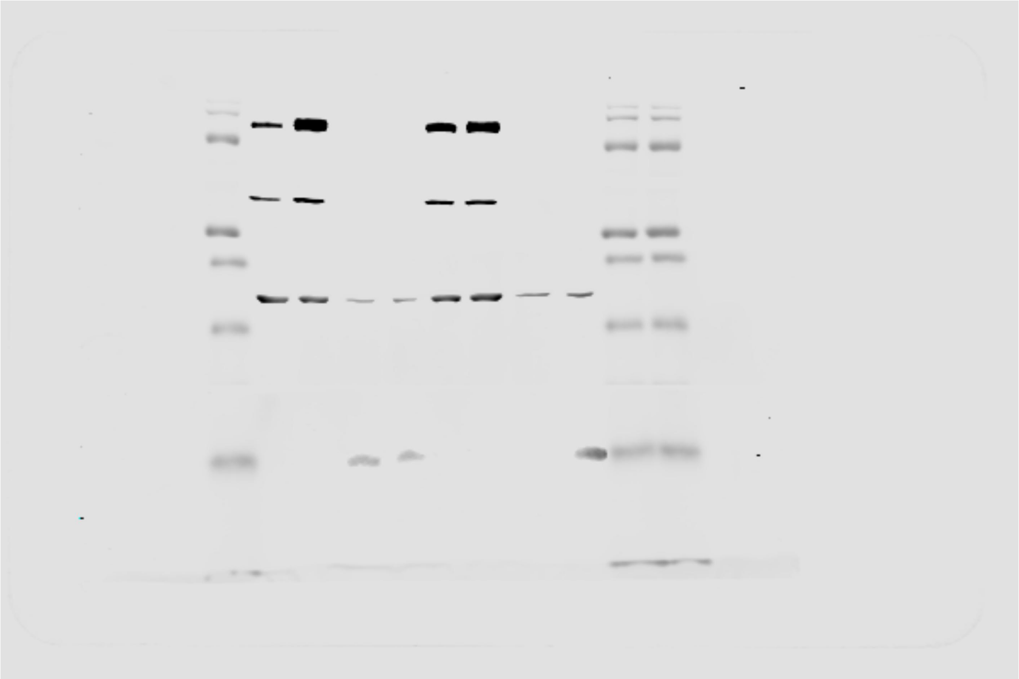

CSQT-2-GAPDH+Histone H3

Fig.S1

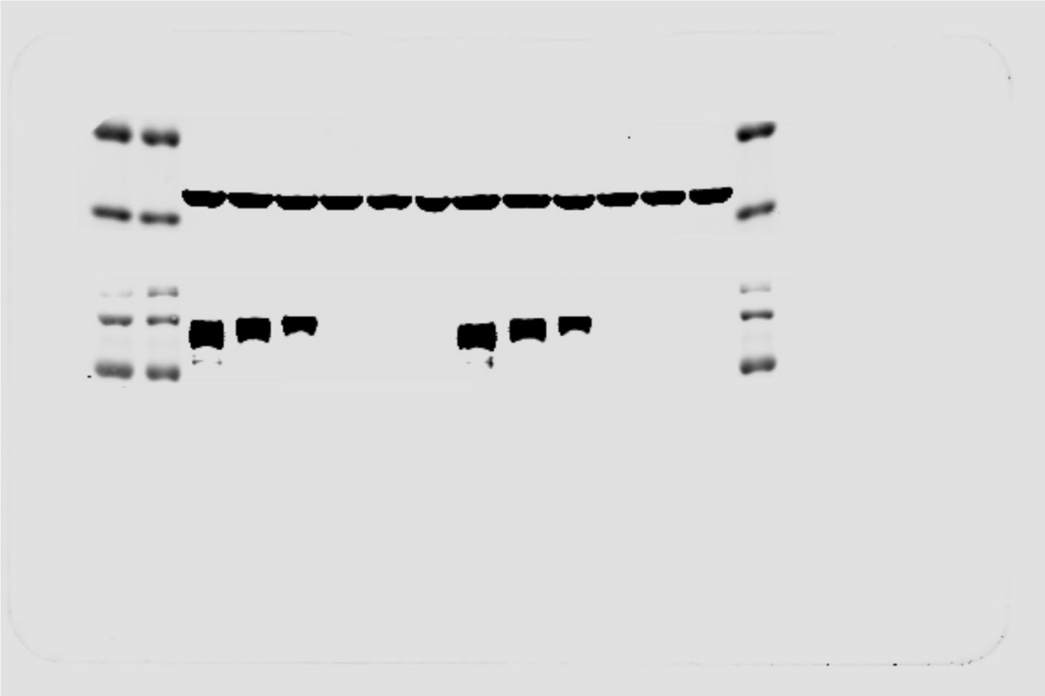

CD146+β-Actin

Fig.S5C

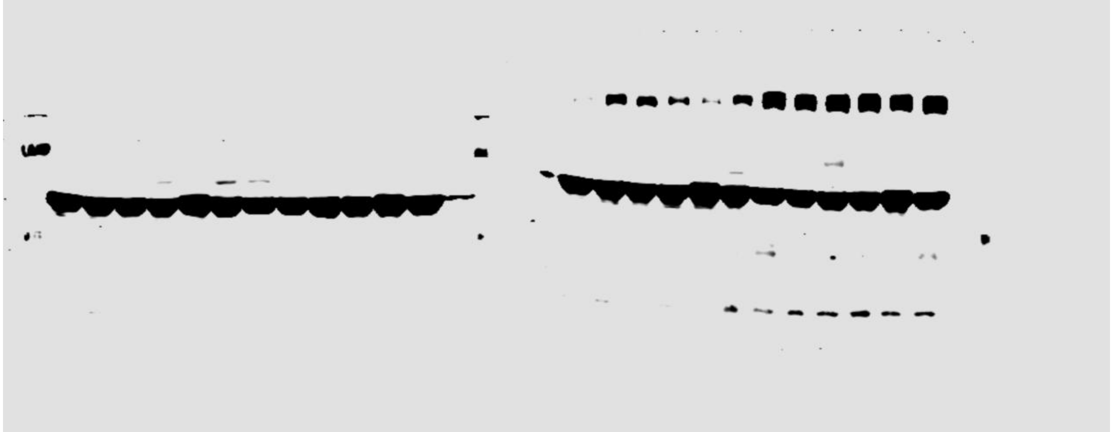

CSQT-2-CD146-HES1

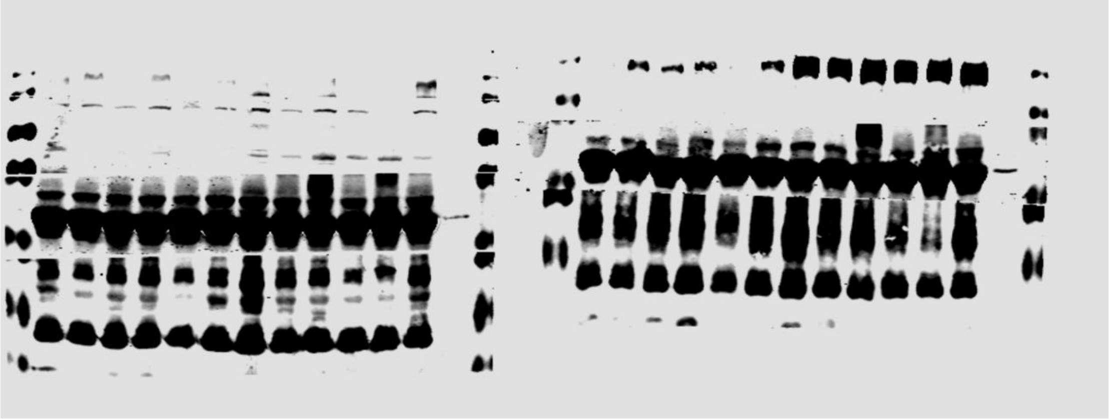

CSQT-2-Notch1

Fig.S5E

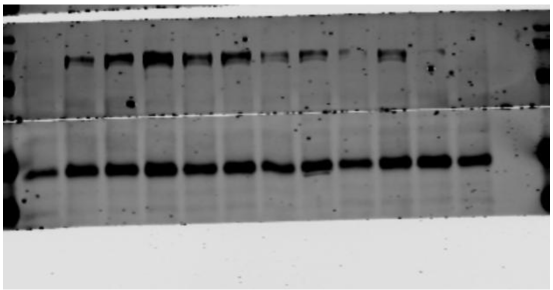

CD146 (PLC)

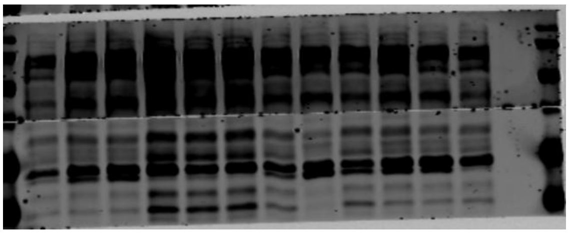

HES1 (PLC)

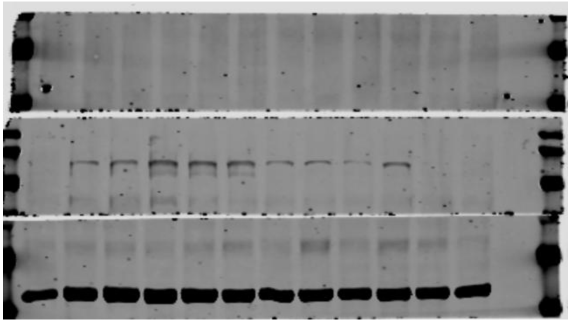

Notch1 (PLC)

Fig.S6B

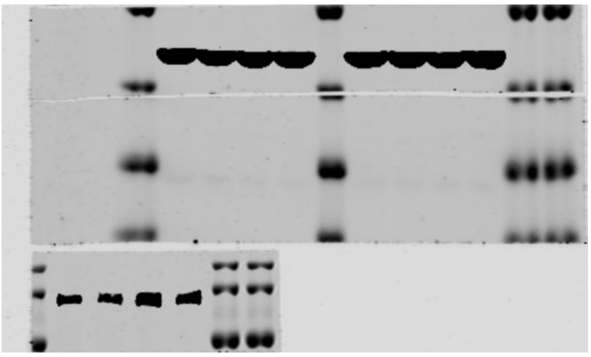

PLC+JAG2 (CD146)
